# A WHO global research priority agenda for wasting and nutritional oedema in infants and children under 5 years

**DOI:** 10.1136/bmjgh-2025-021214

**Published:** 2026-03-23

**Authors:** Allison I Daniel, Jaden Bendabenda, Michael McCaul, Celeste E Naude, Marina Adrianopoli, Zita Weise Prinzo

**Affiliations:** 1 Consultant to the World Health Organization, Geneva, Switzerland; 2 Department of Nutrition and Food Safety, World Health Organization, Geneva, Switzerland; 3 Centre for Evidence-based Health Care, Division of Epidemiology and Biostatistics, Department of Global Health, Stellenbosch University, Cape Town, South Africa

**Keywords:** Decision Making, Global Health, Child health, Nutrition, Study design

## Abstract

**Introduction:**

The guideline development process for the WHO guideline on prevention and management of wasting and nutritional oedema highlighted extensive evidence gaps. We, the WHO Steering Committee and methodologists for the 2023 WHO guideline, therefore aimed to develop a comprehensive global research priority agenda for wasting and nutritional oedema in infants and children. It has a timeframe up to 2030 aligning with the Sustainable Development Goals and Global Nutrition Targets related to wasting and nutritional oedema.

**Methods:**

We used a Child Health and Nutrition Research Initiative (CHNRI) exercise to develop this research priority agenda for four populations and topics of interest: (1) infants less than 6 months of age at risk of poor growth and development; (2) infants and children 6–59 months of age with severe wasting and/or nutritional oedema; (3) infants and children 6–59 months of age with moderate wasting and (4) prevention of wasting and nutritional oedema. For this CHNRI process, we conducted two anonymous surveys, the first to ensure the list of research questions was comprehensive and clear, and the second to score research questions based on their answerability, effectiveness, deliverability and effects on equity.

**Results:**

63 people from 28 countries completed survey 1 and 50 people from 23 countries completed survey 2. We identified 10 priority research questions for each of the four populations and topics of interest, which had median research priority scores of 89.9 (IQR 2.8) and average expert agreement scores with a median of 83.4 (IQR 4.5) indicating high agreement. The research questions are largely focused on delivery and effectiveness of interventions for prevention and management of wasting and nutritional oedema rather than discovery or development.

**Conclusions:**

This research priority agenda will guide researchers and research institutions, funders and others to address pressing research questions on wasting and nutritional oedema.

**WHAT IS ALREADY KNOWN ON THIS TOPIC**

- Addressing wasting and nutritional oedema in infants and children requires prioritisation of key areas for research, particularly considering the many evidence gaps and recent funding reductions.

**WHAT THIS STUDY ADDS**

- We, the WHO Steering Committee and methodologists for the 2023 WHO guideline on wasting and nutritional oedema, coordinated a Child Health and Nutrition Research Initiative (CHNRI) exercise to produce a global research priority agenda for wasting and nutritional oedema through to 2030.
- Top research questions related to evaluating different ready-to-use therapeutic food protocol options with reducing quantities for severe wasting and nutritional oedema; determining reliable criteria for defining optimal growth in infants less than 6 months of age at risk of poor growth and development; assessing cost and cost-effectiveness of specially formulated foods and other dietary interventions for moderate wasting; and identifying effective interventions in humanitarian contexts to prevent wasting and nutritional oedema.

**HOW THIS STUDY MIGHT AFFECT RESEARCH, PRACTICE OR POLICY**

- This research priority agenda will guide researchers, research institutes, funders and policy-makers, among others, to focus their efforts on the most urgent evidence gaps for preventing and managing wasting and nutritional oedema.

## Introduction

 An estimated 42.8 million children worldwide are afflicted by wasting at any given time, equating to a 6.6% prevalence meaning that we are off track to achieve global wasting targets in infants and children under 5 years.^1^,^4^ Global hunger, conflict, climate-related shocks and reductions in funding for nutrition have and will continue to affect efforts to reduce wasting and nutritional oedema and associated deaths, which require urgent action.^5^,^8^

In 2019, WHO committed to updating normative guidance as part of the Global Action Plan (GAP) on Child Wasting to support governments on the prevention and management of wasting and nutritional oedema.^9^ WHO agreed to coordinate and oversee the generation of new evidence and accelerate the process to update normative guidance, ensuring that guidelines reflect the latest evidence on identification and risk characterisation, management and prevention of wasting and nutritional oedema in infants and children.^9^

The *WHO guideline on the prevention and management of wasting and nutritional oedema (acute malnutrition) in infants and children under 5 years* was published in 2023, addressing 16 guideline questions that were prioritised by the Guideline Development Group (GDG).^10^ The guideline scope included four populations and topics of focus: (1) infants less than 6 months of age at risk of poor growth and development, (2) infants and children 6–59 months of age with severe wasting and nutritional oedema, infants and children 6–59 months of age with moderate wasting and (4) prevention of wasting and nutritional oedema.

Many of the guideline questions prioritised for the guideline had limited evidence and/or very low or low certainty evidence based on the GRADE (Grading of Recommendations, Assessment, Development and Evaluations) approach,^11 12^ which the GDG examined and used to inform recommendations. There were also guideline questions and interventions for which the GDG did not make recommendations, mainly due to a lack of directly relevant evidence. Furthermore, there are additional priority areas beyond the guideline questions addressed in the 2023 WHO guideline that were not included in the list of 16 initial guideline questions. This list of guideline questions was generated based on the findings of four scoping reviews, current WHO guidance, inputs from collaborators and knowledge-users (WHO Member States and implementing organisations) and previous WHO technical consultation meetings (preprioritisation stage).^13^

Throughout the guideline development process, the GDG identified many research needs related to the guideline questions under consideration, which were captured and listed in the guideline.^10^ However, this lengthy list was not established using systematic methods and does not indicate the areas of research that should be prioritised.

As indicated in the GAP,^9^ WHO therefore aimed to develop a comprehensive global research priority agenda for wasting and nutritional oedema in infants and children under 5 years, building on the 2023 WHO guideline with plans to update the guideline as new evidence emerges. This research priority agenda has a goal of addressing critical evidence gaps related to child health and health equity in these populations. The timeline of this agenda will extend through to 2030. This is in line with the Sustainable Development Goals and the Global Nutrition Targets, which have been extended to 2030.^3 4 14^

## Methods

We, the WHO Steering Committee and methodologists for the 2023 WHO guideline on wasting and nutritional oedema, coordinated the development of this global research priority agenda using a Child Health and Nutrition Research Initiative (CHNRI) exercise and in accordance with WHO guidance on research priority setting.^15^,^17^ The CHNRI method is a systematic approach for setting research priorities, involving independent scoring of research questions based on a predefined set of criteria.^15^,^17^

Our team members have a content focus on wasting and nutritional oedema, as well as methods and research expertise including using evidence and a formalised, structured and transparent process for guideline development.^18^

We used the REporting guideline for PRIority SEtting of health research framework for reporting.^19^

### Scope and context for the research priority agenda

This research priority agenda is global in scope. It has a timeframe for research up to 2030 aligning with the global goals related to wasting and nutritional oedema outlined above. The target audience for this research priority agenda includes but is not limited to researchers and research institutions, as well as policy makers and funders who we aim to engage about the uptake and utility of this research priority agenda.

As with the 2023 WHO guideline, this research priority agenda has a child health perspective, with children’s health, growth and development at the forefront, as opposed to a public health approach. This was one of the guiding principles that served as the foundation of the 2023 WHO guideline.^20^ It also takes an explicit health equity lens with the goal of prioritising research that can reduce health inequities and improve outcomes in disadvantaged children and their families.

The populations and topics of interest for this research priority agenda, aligning with the 2023 WHO guideline, include:

1. Infants less than 6 months of age at risk of poor growth and development.
2. Infants and children aged 6–59 months with severe wasting and/or nutritional oedema.
3. Infants and children aged 6–59 months with moderate wasting.
4. Prevention of wasting and nutritional oedema.

### Participants in the CHNRI process

We aimed to include a representative group of experts in the prevention and management of wasting and nutritional oedema, to participate in this CHNRI process that involved two surveys. These could include any researchers, clinicians, policy-makers, programme implementers and community partners with expertise in wasting and nutritional oedema. We shared the two surveys for the CHNRI process with the UNICEF/WHO Technical Advisory Group on Wasting and Nutritional Oedema, the GDG and observers for the 2023 WHO guideline, team leads of systematic reviews commissioned for the 2023 WHO guideline, and attendees of a 2024 research consultation on wasting and nutritional oedema. When sharing the survey, we also asked these invitees to circulate the survey widely. WHO regional advisers disseminated the survey as well. We also posted the surveys publicly on the Emergency Nutrition Network forum (https://www.en-net.org/). There were no incentives to complete the surveys; participation was voluntary.

### Research questions from the 2023 WHO guideline

As a first step, we consolidated all the research needs listed in the 2023 WHO guideline for each respective guideline question, which emerged from GDG discussions throughout the guideline development process, and converted these to questions. We categorised these research questions according to the four populations and topics described above, and further organised them by research subdomains adapted for nutrition by Lelijveld *et al*,^21^ including descriptive, delivery, development and discovery, as defined here:

- Descriptive: research to assess the burden of the problem, its determinants and effectiveness of interventions to address the problem.
- Delivery: research to improve how nutrition interventions are delivered, financed and taken up.
- Development: research to improve nutrition interventions that already exist.
- Discovery: research that leads to innovation, that is, entirely new nutrition interventions.

### Surveys and scoring within the CHNRI process

Between February and June 2025, potential participants were invited to complete two survey rounds anonymously (online supplemental file 1). Survey 1 was open for 4 weeks initially and was extended for an additional 2 weeks; survey 2 was open for 6 weeks and was extended for an additional 2 weeks. Participants were allowed to participate in either or both rounds.

We collected basic descriptive information about participants including gender, location, expertise and institution in each of the two rounds. We asked participants to include their email address in the round 1 survey if they would like to receive the round 2 survey, which was kept separate from all other survey results to maintain anonymity.

#### Survey 1

Survey 1 was undertaken to ensure the list of research questions was comprehensive and clear for scoring within survey 2. For the round 1 survey, we shared the prepared list of research questions from the 2023 WHO guideline as described above. The objectives of survey 1 were:

1. To identify potential research questions that will enhance understanding of wasting and nutritional oedema and have the potential to inform policy and practice.
2. To ensure the list of research questions is comprehensive and clear.
3. To collect expert feedback and suggestions for improvement.

We invited participants to add any additional priority research questions that had not already been included. They could add research questions if they felt that these additional questions would have the potential to enhance understanding of wasting and nutritional oedema and/or could inform policy. There was no limit to the number of research questions that could be proposed by participants.

Participants could also provide feedback or suggestions to improve the clarity or framing of any research questions, including specific suggestions for wording changes, additional context or reorganisation of the research questions. We requested that participants submit the survey even if they had no additional proposed research questions or comments.

We then reviewed the responses to revise the list of questions and to identify new questions for inclusion in the list. Only unique research questions that were within scope of the four populations and topics of interest were added to the list of questions for scoring in survey 2. If proposals were not added in a question format, we converted these to research questions.

#### Survey 2

The objective of survey 2 was to score research questions based on four CHNRI criteria: answerability, effectiveness, deliverability and effects on equity^15 16^ as follows:

- Answerability: Research studies (qualitative and/or quantitative) to answer this research question are feasible and ethical, and can be done by 2030.
- Effectiveness: The findings of this research (qualitative and/or quantitative) could lead to effective interventions, programmes or policies that improve child health.
- Deliverability: The findings of this research (qualitative and/or quantitative) could lead to interventions, programmes, or policies that can realistically be implemented.
- Effect on equity: The findings of this research (qualitative and/or quantitative) could contribute to reducing health inequities among disadvantaged children.

The rationale for selecting these criteria was that they reflect a focus on the child health perspective that was a guiding principle in the development of the 2023 WHO guideline, described earlier.^20^ These criteria may also lead to the generation of evidence that could inform WHO recommendations, including that around effectiveness and equity, as well as implementation of recommendations and good practice statements reflected in the deliverability criterion.

According to Rudan *et al*,^15^ these four criteria, along with ‘maximum potential for disease burden reduction’, are recommended for use in most CHNRI exercises. We did not include the criterion around disease burden reduction, as our aim was to identify research questions that will ultimately strengthen prevention and management of wasting and nutritional oedema.

We deliberately excluded other criteria, such as cost and feasibility,^15^ as costs of conducting research may vary substantially across settings and contexts, therefore limiting applicability for this global research priority agenda. Furthermore, there may be a need for investment into high quality research to answer priority research questions.

In survey 2, participants were first asked to select the population(s) and topic(s) for which they wanted to score each research question based on their expertise. We asked that participants read each research question carefully, reflecting on its importance and relevance to the identified population(s) and topic(s) of interest. They then scored the research questions according to the four CHNRI criteria above, with the options being ‘agree,’ ‘neither agree nor disagree,’ ‘disagree,’ and ‘don’t know’.

In terms of scoring, ‘agree’ counted as 1 point; ‘neither agree nor disagree’ counted as 0.5 points; and ‘disagree’ counted for no points. ‘Don’t know’ was not counted as a recorded score, with any ‘don’t know’ responses omitted from the numerator and denominator. Note that all questions were mandatory if respondents agreed to score a certain population or topic; they could not advance if a question was left blank.

We calculated research priority scores (RPSs) of between 0 and 100 for each research question by adding up the scores for each of the four criteria, which were weighted equally, divided by the number of answers and then averaging across the four criteria. The top 20 research questions overall based on RPSs, as well as the top 10 research questions for each of the four populations or topics based on RPSs, were displayed as priority areas for research. Average expert agreement (AEA) scores were calculated and presented alongside RPSs, with scores closer to 100 indicating high agreement. Specifically, AEA is the proportion of experts giving the mode score for each research question relative to the total number of responses for that particular research question, excluding the ‘don’t know’ answers.

### Patient and public involvement

Patients and caregivers were not involved in this CHNRI process.

## Results

63 participants across 28 countries completed survey 1 and 50 participants across 23 countries completed survey 2. There were participants from all WHO regions for both surveys, with almost one-third being from the African Region for both surveys. There were few respondents from the Western Pacific Region. Most respondents were researchers, making up 60.3% for survey 1 and 78.0% for survey 2. There was a high proportion of programme implementers for survey 1 in particular (46.0%). In terms of the organisation types, many participants are based at academic/research institutions (31.7% for survey 1 and 42.0% for survey 2) and non-governmental organisations (38.1% for survey 1 and 40.0% for survey 2). Further participant information is shown in table 1.

**Table 1:** Participant information for each of the two surveys

	Survey 1, n (%)	Survey 2, n (%)
Gender		
Woman Man Non-binary Other Prefer not to answer	35 (56%) 25 (40%) 0 0 3 (5%)	30 (60%) 19 (38%) 0 0 1 (2%)
Primary location (WHO region)		
African Region Eastern Mediterranean Region European Region Region of the Americas South-East Asian Region Western Pacific Region	20 (32%) 6 (10%) 18 (29%) 10 (16%) 8 (13%) 1 (2%)	16 (32%) 5 (10%) 11 (22%) 9 (18%) 7 (14%) 2 (4%)
Role/profession/expertise*		
Researcher Clinician Policy-maker Programme implementer Community partner Other	38 (60%) 12 (19%) 3 (5%) 29 (46%) 2 (3%) 7 (11%)	39 (78%) 6 (12%) 4 (8%) 12 (24%) 2 (4%) 7 (14%)
Organisation type*		
Academic/research institution Health facility Health ministry United Nations agency Non-governmental organisation Community-based organisation Independent Other	20 (32%) 4 (6%) 2 (3%) 14 (22%) 24 (38%) 1 (2%) 4 (6%) 5 (8%)	21 (42%) 5 (10%) 0 5 (10%) 20 (40%) 1 (2%) 3 (6%) 6 (12%)

* Participants could select multiple options.

### List of research questions for scoring

The number of research questions that were identified from the 2023 WHO guideline process and following survey 1 is summarised in table 2. There were eight additional questions proposed for infants less than 6 months of age at risk of poor growth and development; 32 for infants and children 6–59 months of age with severe wasting and/or nutritional oedema; 13 for infants and children 6–59 months of age with moderate wasting; and 10 for prevention of wasting and nutritional oedema.

**Table 2:** Number of research questions identified from the 2023 WHO guideline and after survey 1

Population or topic	Number of research questions from the 2023 WHO guideline	Number of research questions after survey 1
Infants less than 6 months of age at risk of poor growth and development	25	33
Infants and children 6–59 months of age with severe wasting and/or nutritional oedema	30	62
Infants and children 6–59 months of age with moderate wasting	16	29
Prevention of wasting and nutritional oedema	16	26

### Prioritisation of research questions after scoring based on CHNRI criteria

**Figure 1:**
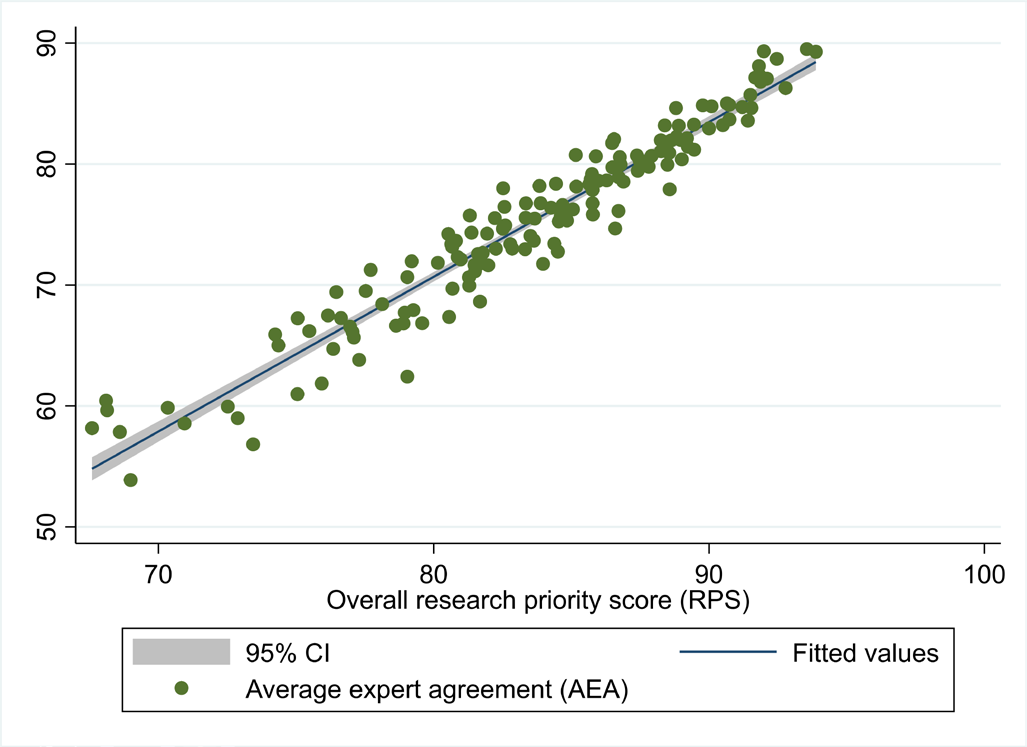
Relationship between research priority scores and average expert agreement.

For the 150 research questions that could be scored, the median number of responses excluding blanks and ‘don’t know’ answers was 33 (IQR 3). RPS ranged from 67.6 to 93.9 and AEA ranged from 53.9 to 89.5. There was a strong correlation between RPS and AEA with linear regression showing a one-point increase in RPS was associated with a 1.28-point increase in AEA (95% CI 1.22 to 1.34) as shown in figure 1.

The top 20 research questions across the four populations and topics of interest based on RPS are shown in table 3. The median RPS for these 20 questions was 91.6 (IQR 1.3) with a median AEA of 86.0 (IQR 2.9). There were five research questions on infants less than 6 months of age at risk of poor growth and development; five on infants and children 6–59 months of age with severe wasting and/or nutritional oedema; seven on infants and children 6–59 months of age with moderate wasting; and three on prevention of wasting and nutritional oedema. Thirteen of the 20 top research questions were in the subdomain delivery, six in the subdomain description, one in the subdomain development and none in the subdomain discovery.

**Table 3:** Overall top 20 research questions for wasting and nutritional oedema across the four populations and topics of interest

Ranking	Population or topic	Research question	Research subdomain	RPS	AEA
1	Infants and children 6–59 months of age with severe wasting and/or nutritional oedema	What is the effectiveness of different ready-to-use therapeutic food (RUTF) protocol options with reducing quantities compared with standard quantities?	Delivery	93.9	89.3
2	Infants and children 6–59 months of age with severe wasting and/or nutritional oedema	What are potential risk-targeted follow-up strategies of infants and children with severe wasting and/or nutritional oedema?	Delivery	93.6	89.5
3	Infants less than 6 months of age at risk of poor growth and development	What are reliable criteria for defining optimal growth in infants less than 6 months of age at risk of poor growth and development?	Description	92.8	86.3
4	Infants and children 6–59 months of age with moderate wasting	What are the cost and cost-effectiveness of specially formulated foods (SFFs) and other dietary interventions?	Delivery	92.5	88.7
5	Prevention of wasting and nutritional oedema	What are the most effective interventions in humanitarian contexts to prevent wasting and nutritional oedema?	Description	92.1	87.1
6	Infants and children 6–59 months of age with moderate wasting	What is the cost-effectiveness of integrating moderate wasting into the primary healthcare system (in places where moderate wasting is managed outside the health system)?	Delivery	92.0	89.3
7	Infants and children 6–59 months of age with severe wasting and/or nutritional oedema	What tools, support systems, etc can be used to maximise effectiveness of community health workers management?	Delivery	91.9	86.8
8	Infants and children 6–59 months of age with moderate wasting	Which infants and children with moderate wasting should be prioritised for supplementation based on risk factors?	Delivery	91.8	87.4
9	Infants and children 6–59 months of age with severe wasting and/or nutritional oedema	What are the cost and cost-effectiveness of different quantities of RUTF?	Delivery	91.8	88.1
10	Infants and children 6–59 months of age with moderate wasting	Which interventions are effective to prevent relapse and improve long-term health and development outcomes in infants and children with moderate wasting?	Description	91.7	87.1
11	Prevention of wasting and nutritional oedema	What are the cost and cost-effectiveness of preventive interventions for wasting and nutritional oedema compared with each other?	Delivery	91.5	84.6
12	Infants less than 6 months of age at risk of poor growth and development	Which tools and strategies can health workers use to assess and manage simple breastfeeding problems effectively?	Delivery	91.5	85.7
13	Infants and children 6–59 months of age with moderate wasting	Would infants and children with moderate wasting who do not meet criteria for prioritisation with SFFs in low-risk settings benefit from a dietary intervention?	Description	91.4	83.6
14	Infants less than 6 months of age at risk of poor growth and development	What intervention packages to support existing health staff, such as using peer counsellors for the assessment and management of breastfeeding/lactation difficulties, exist and how do they compare?	Development	91.2	84.7
15	Infants and children 6–59 months of age with moderate wasting	What are the most effective and cost-effective targeting strategies for the provision of SFFs for moderate wasting in humanitarian settings?	Delivery	90.7	83.7
16	Infants less than 6 months of age at risk of poor growth and development	What is the effectiveness of different types of formulas including F-75, diluted F-100, full-strength F-100, and infant formulas (potentially consider pre-term formulas if appropriate) as well as donor human milk in inpatient settings for infants less than 6 months of age at risk of poor growth and development, as well as those with severe wasting and/or nutritional oedema specifically?	Description	90.7	84.9
17	Infants and children 6–59 months of age with moderate wasting	What are the cost and cost-effectiveness of managing moderate wasting using available home foods in different contexts?	Delivery	90.6	85.0
18	Infants less than 6 months of age at risk of poor growth and development	How should infants less than 6 months of age at risk of poor growth and development who fail to respond to initial supported breastfeeding and clinical treatment be managed?	Delivery	90.5	83.2
19	Infants and children 6–59 months of age with severe wasting and/or nutritional oedema	Which interventions are effective to prevent relapse and improve long-term health and development outcomes in infants and children with severe wasting and/or nutritional oedema?	Description	90.1	84.8
20	Prevention of wasting and nutritional oedema	What is the cost-effectiveness of interventions for prevention of wasting and nutritional oedema, including logistics and implementation costs?	Delivery	90.0	83.3

AEA, average expert agreement; RPS, research priority score.

The top 10 research questions for infants less than 6 months of age at risk of poor growth and development (table 4), which were scored by 38 participants in survey 2, had a median RPS of 89.7 (IQR 2.5) and median AEA of 83.9 (IQR 2.9). Four of these were description questions, four delivery questions and two development questions, with no discovery questions. The top question focused on areas such as reliable criteria for defining optimal growth and development, with other priority research questions also focusing on identifying and managing these infants. There were additional research questions focused on breastfeeding support and specifically tools and strategies and intervention packages to support existing health staff. In addition, there were research questions about the effectiveness of different types of formulas including but not limited to therapeutic milks, as well as donor human milk and wet nursing.

**Table 4:** Top 10 research questions for infants less than 6 months of age at risk of poor growth and development

Ranking	Research question	Research subdomain	RPS					AEA
			A	E	D	EE	Overall	
1	What are reliable criteria for defining optimal growth in infants less than 6 months of age at risk of poor growth and development?	Description	91.4	94.1	90.0	95.6	92.8	86.3
2	Which tools and strategies can health workers use to assess and manage simple breastfeeding problems effectively?	Delivery	90.5	91.9	91.9	91.7	91.5	85.7
3	What intervention packages to support existing health staff, such as using peer counsellors for the assessment and management of breastfeeding/lactation difficulties, exist and how do they compare?	Development	92.4	93.9	89.4	89.1	91.2	84.7
4	What is the effectiveness of different types of formulas including F-75, diluted F-100, full-strength F-100 and infant formulas (potentially consider preterm formulas if appropriate) as well as donor human milk in inpatient settings for infants less than 6 months of age at risk of poor growth and development, as well as those with severe wasting and/or nutritional oedema specifically?	Description	94.6	94.4	90.5	83.3	90.7	84.9
5	How should infants less than 6 months of age at risk of poor growth and development who fail to respond to initial supported breastfeeding and clinical treatment be managed?	Delivery	91.2	94.1	91.4	85.3	90.5	83.2
6	What screening criteria should be used to detect infants less than 6 months of age at risk of poor growth and development in the community?	Description	94.7	88.2	89.5	82.9	88.8	82.2
7	Which packages of care are most effective at improving outcomes in infants less than 6 months of age at risk of poor growth and development and preventing wasting and nutritional oedema in these infants?	Development	90.3	89.2	87.5	88.2	88.8	84.6
8	What are the acceptability and existing practices around wet nursing, supplementary suckling technique, re-establishment of breastfeeding, etc?	Description	90.3	88.9	90.0	84.7	88.5	81.8
9	What prioritisation criteria can be used for infants less than 6 months of age at risk of poor growth and development if caseloads are high and resources limited?	Delivery	88.6	88.2	87.1	89.7	88.4	81.2
10	What are the priority medical and nutritional support and interventions for mothers/caregivers of infants less than 6 months of age at risk of poor growth and development?	Delivery	87.5	90.5	84.7	88.9	87.9	80.7

A, answerability; AEA, average expert agreement; D, deliverability; E, effectiveness; EE, effect on equity; RPS, research priority score.

For the top 10 research questions for infants and children 6–59 months of age with severe wasting and nutritional oedema (table 5), the median RPS was 89.6 (IQR 2.9) and the median AEA was 84.0 (IQR 6.6), scored by 34 participants. Most of these questions were on delivery (seven in total), while three were description questions; none were development or discovery questions. There were multiple research questions around the effectiveness, cost and cost-effectiveness of different ready-to-use-therapeutic food (RUTF) approaches with reducing quantities. Additional questions focused on risk-targeted follow-up strategies, risk factors for relapse to severe wasting and/or nutritional oedema, and prevention of relapse and improved long-term outcomes. Two additional questions related to the effectiveness and cost-effectiveness, respectively, of community health workers (CHWs) in the management of severe wasting and/or nutritional oedema.

**Table 5:** Top 10 research questions for infants and children 6–59 months of age with severe wasting and/or nutritional oedema

Ranking	Research question	Research subdomain	RPS					AEA
			A	E	D	EE	Overall	
1	What is the effectiveness of different ready-to-use therapeutic food (RUTF) protocol options with reducing quantities compared with standard quantities?	Delivery	92.4	93.9	97.0	92.2	93.9	89.3
2	What are potential risk-targeted follow-up strategies of infants and children with severe wasting and/or nutritional oedema?	Delivery	96.9	95.5	88.3	95.5	93.6	89.5
3	What tools, support systems, etc can be utilised to maximise effectiveness of community health workers (CHWs) management?	Delivery	90.9	92.2	92.2	92.2	91.9	86.8
4	What are the cost and cost-effectiveness of different quantities of RUTF?	Delivery	95.6	92.6	92.6	86.4	91.8	88.1
5	Which interventions are effective to prevent relapse and improve long-term health and development outcomes in infants and children with severe wasting and/or nutritional oedema?	Description	87.5	95.6	87.9	89.4	90.1	84.8
6	What chronic conditions are associated with non-response or treatment failure in infants and children with severe wasting and/or nutritional oedema?	Description	92.6	89.7	81.8	92.6	89.2	82.1
7	What are the cost and cost-effectiveness of alternative RUTF formulations in different settings?	Delivery	92.2	92.4	85.9	85.4	89.0	80.4
8	What are the risk factors (individual, household, community, programme-level) for relapse to severe wasting and/or nutritional oedema?	Description	92.4	86.4	84.4	92.4	88.9	83.2
9	What is the effectiveness of CHWs in management of severe wasting and/or nutritional oedema throughout the care pathway?	Delivery	84.8	89.4	89.1	90.9	88.6	80.9
10	What is the cost-effectiveness of CHWs for management of severe wasting and/or nutritional oedema and the impacts of this approach on coverage and other services?	Delivery	84.8	89.4	89.1	90.9	88.6	80.9

A, answerability; AEA, average expert agreement; D, deliverability; E, effectiveness; EE, effect on equity; RPS, research priority score.

The top 10 research questions for infants and children 6–59 months of age with moderate wasting (table 6) were based on scoring done by 34 participants, with a median RPS of 91.1 (IQR 1.8) and median AEA of 84.9 (IQR 3.7). As with severe wasting and nutritional oedema, seven were delivery questions and three were description questions. There were no development or discovery questions in the top 10. Several questions focused on effectiveness, cost and cost-effectiveness of dietary interventions including but not limited to specially formulated foods (SFFs). There were two questions related to which children to prioritise for supplementation based on risk factors and whether children not prioritised would benefit from a dietary intervention, respectively. Two questions emerged on relapse, including risk factors for relapse to moderate wasting and which interventions are effective to prevent relapse and improve long-term outcomes. In addition, there were multiple questions on CHW management and integration into the primary healthcare system.

**Table 6:** Top 10 research questions for infants and children 6–59 months of age with moderate wasting

Ranking	Research question	Research subdomain	RPS					AEA
			A	E	D	EE	Overall	
1	What are the cost and cost-effectiveness of specially formulated foods (SFFs) and other dietary interventions?	Delivery	98.5	94.1	89.7	87.5	92.5	88.7
2	What is the cost-effectiveness of integrating moderate wasting into the primary healthcare system (in places where moderate wasting is managed outside the health system)?	Delivery	93.9	93.9	87.9	92.2	92.0	89.3
3	Which infants and children with moderate wasting should be prioritised for supplementation based on risk factors?	Delivery	92.6	97.1	89.7	87.9	91.8	87.4
4	Which interventions are effective to prevent relapse and improve long-term health and development outcomes in infants and children with moderate wasting?	Description	89.4	98.5	86.4	92.4	91.7	87.1
5	Would infants and children with moderate wasting who do not meet criteria for prioritisation with SFFs in low-risk settings benefit from a dietary intervention?	Description	95.6	95.6	83.8	90.6	91.4	83.6
6	What are the most effective and cost-effective targeting strategies for the provision of SFFs for moderate wasting in humanitarian settings?	Delivery	86.8	94.1	91.2	90.9	90.7	83.7
7	What are the cost and cost-effectiveness of managing moderate wasting using available home foods in different contexts?	Delivery	91.2	91.2	91.2	89.1	90.6	85.0
8	What is the effectiveness of community health workers (CHWs) in management of moderate wasting throughout the care pathway?	Delivery	87.9	92.4	86.4	92.4	89.8	84.8
9	What are the risk factors (individual, household, community, programme-level) for relapse to moderate wasting?	Description	95.6	89.7	86.8	84.8	89.2	81.4
10	What tools, support systems, etc can be used to maximise effectiveness of CHWs management?	Delivery	87.5	89.1	84.8	93.5	88.6	82.0

A, answerability; AEA, average expert agreement; D, deliverability; E, effectiveness; EE, effect on equity; RPS, research priority score.

35 participants scored the research questions for prevention of wasting and nutritional oedema, with the top 10 questions (table 7) having a median RPS of 89.2 (IQR 2.2) and median AEA 81.6 (IQR 4.0). Three were description questions, five were delivery questions and two were development questions. There were no discovery questions. The top question was around determining the most effective interventions for prevention in humanitarian contexts. Other questions related to cost and cost-effectiveness of preventive interventions, as well as direct, indirect and opportunity costs to families of food-based interventions. Additional questions related to food-based interventions, cash programming, maternal interventions and scalable livelihood initiatives.

**Table 7:** Top 10 research questions for prevention of wasting and nutritional oedema

Ranking	Research question	Research subdomain	RPS					AEA
			A	E	D	EE	Overall	
1	What are the most effective interventions in humanitarian contexts to prevent wasting and nutritional oedema?	Description	85.7	97.1	88.6	97.0	92.1	87.1
2	What are the cost and cost-effectiveness of preventive interventions for wasting and nutritional oedema compared with each other?	Delivery	90.6	92.2	87.9	95.5	91.5	84.6
3	What is the cost-effectiveness of interventions for prevention of wasting and nutritional oedema, including logistics and implementation costs?	Delivery	92.6	88.2	89.7	89.4	90.0	83.3
4	What are the most effective and cost-effective platforms or services to implement food-based interventions for prevention of wasting and nutritional oedema?	Delivery	89.7	91.2	87.9	89.1	89.5	81.2
5	What is the potential role of cash programming in prevention of wasting and nutritional oedema, in isolation or in combination with other interventions?	Development	95.6	89.7	84.3	88.2	89.5	83.3
6	What low-cost and scalable livelihood initiatives can build resilience against wasting and nutritional oedema?	Development	83.9	90.3	88.3	93.3	89.0	82.0
7	What is the effectiveness of other types of prevention interventions apart from small-quantity lipid-based nutrient supplements (SQ-LNS) and medium-quantity lipid-based nutrient supplements (MQ-LNS) for prevention of wasting and nutritional oedema?	Description	89.4	94.1	84.8	85.9	88.6	77.9
8	What are the optimal quantity, duration and timing of food-based interventions for prevention of wasting and nutritional oedema?	Delivery	89.7	92.6	83.8	83.3	87.4	80.7
9	What are the direct, indirect and opportunity costs to families of food-based interventions for prevention of wasting and nutritional oedema?	Delivery	86.4	85.3	85.3	89.4	86.6	74.7
10	What are the potential impacts of maternal interventions in the context of prevention of wasting and nutritional oedema?	Description	87.9	88.2	81.4	86.4	86.0	78.6

A, answerability; AEA, average expert agreement; D, deliverability; E, effectiveness; EE, effect on equity; RPS, research priority score.

## Discussion

We coordinated the development of this research priority agenda to give guidance to the research community and funders for prioritising research that will support a stronger evidence base for prevention and management of wasting and nutritional oedema in infants and children.

We used the 2023 WHO guideline as a foundation for developing this research priority agenda. Colleagues at WHO have previously suggested that guideline development processes provide ‘a unique and efficient opportunity to compile an agenda from the research needs identified by each of the GDG’.^22^

### Research questions classified by subdomain

Of the overall top research questions across the four populations and topics of interest, a majority (13/20) were delivery questions. This points to gaps in our knowledge around how to strengthen interventions on the prevention and management of wasting and nutritional oedema in terms of their provision and implementation (e.g. coordination and management of care processes, how and when interventions and care are delivered), some of which also centre on or include financing and coverage. Six of the top 20 research questions were description questions and specifically relevant to better understanding the effectiveness of interventions for prevention and management. There was only one development question to enhance interventions that exist already and there were no discovery questions in the top 20 across the four populations and topics. This distribution around the research subdomains indicates that the highest priority research questions relate to evaluation, implementation and optimisation of existing interventions, in contrast to novel prevention and management approaches.

#### Infants less than 6 months of age at risk of poor growth and development

Key priority areas for infants less than 6 months of age at risk of poor growth and development were around criteria for defining optimal growth, detecting infants at risk in the community, and prioritisation criteria if caseloads are high and resources limited. This indicates the need to further understand how to characterise this population of infants at risk and how to manage these infants in practice, including in community settings and where there are resource constraints.

In line with these challenges, a recent analysis in Ethiopia explored the potential caseload based on criteria for identifying infants at risk of poor growth and development in the 2023 WHO guideline, estimating it to be around 19.2% in infants 6 weeks to 6 months who visited health centres for ‘delivery, immunisation, growth monitoring and acute disease treatment’.^23^ A survey to evaluate primary healthcare readiness in Senegal for management of mothers and infants less than 6 months of age who are at risk showed that gaps exist in terms of classifying and managing infants at risk as well as equipment and training.^24^

Other high-ranking research questions for infants less than 6 months of age at risk for poor growth and development about breastfeeding support for mothers/caregivers of these infants, including tools and strategies for health workers to assess and manage simple breastfeeding problems effectively, and what to do when infants do not respond to initial supported breastfeeding and clinical treatment. These topics were addressed in the 2023 WHO guideline but had limited evidence for the GDG to develop new recommendations and to update recommendations from the 2013 *WHO Guideline: updates on the management of severe acute malnutrition in infants and children*, which covered infants less than 6 months of age with severe wasting and/or nutritional oedema.^10 25^

A separate commentary led by GDG members and others involved in the 2023 WHO guideline development process highlights many evidence gaps in infants less than 6 months of age at risk of poor growth and development and includes suggestions about how to answer these gaps through research. Gaps include identifying and managing infants at risk of poor growth and development, addressing and managing breastfeeding challenges, indications for use of supplemental milks, and effective maternal-directed interventions.^26^

#### Infants and children aged 6–59 months with severe wasting and/or nutritional oedema

Three overarching themes emerged within the top 10 prioritised research questions for infants and children with severe wasting and/or nutritional oedema including RUTF approaches, CHW management and understanding and preventing relapse. First, there were research questions related to the effectiveness, as well as cost and cost-effectiveness, of different quantities of RUTF. There was also one research question about the cost and cost-effectiveness of alternative RUTF formulations in different settings.

Furthermore, research questions emerged about how to maximise success of management by CHWs. This is relevant to decision-making and implementation of a conditional recommendation in the 2023 WHO guideline indicating that assessment and management of wasting and nutritional oedema in infants and children 6–59 months of age can be done by CHWs ‘as long as they receive adequate training, and regular supervision of their work is built into service delivery’.^10^ This research question was prioritised for both severe wasting and/or nutritional oedema and moderate wasting.

Another commentary led by GDG members and others involved in the 2023 WHO guideline process highlighted evidence gaps around management of severe wasting and/or nutritional oedema.^27^ Two main evidence gaps, which align with the research priorities from this CHNRI process, were around limitations in the evidence to inform risk-based care for improving important outcomes and challenges around implementation and scale-up of CHWs for identification and management in different contexts.^27^

#### Infants and children aged 6–59 months with moderate wasting

The research questions that were prioritised in the top 10 for infants and children with moderate wasting were predominantly around dietary interventions for moderate wasting including but not limited to SFFs. One research question is specifically on the cost and cost-effectiveness of SFFs compared with other dietary intervention and another is about whether children with moderate wasting who do not meet criteria for SFFs would benefit from a dietary intervention in low-risk settings. There was an additional question on the cost and cost-effectiveness of managing moderate wasting using available home foods in different contexts.

These research questions on the use of home foods or other dietary approaches apart from SFFs reflect a lack of evidence around non-SFF approaches for management of moderate wasting as indicated in the 2023 WHO guideline and highlighted in another commentary led by GDG members and others involved in the 2023 WHO guideline.^10 28^ The evidence gaps related to nutritional supplementation approaches, including understanding the impact of improved breastfeeding, home foods and other non-SFF interventions; prioritisation for SFFs; and quantity of SFFs to be provided in infants and children who may need these.^28^

#### Prevention of wasting and nutritional oedema

The top research question for prevention of wasting and nutritional oedema was about the most effective interventions in humanitarian contexts for prevention. One of the guiding principles for the 2023 WHO guideline is about the importance of local adaptation, with a statement that ‘special consideration should be given to how to implement these recommendations in humanitarian crises and the importance of reviewing any adaptations made as crises evolve and/or stabilise’.^10^

Other top 10 research questions on prevention of wasting and nutritional oedema had a strong focus on cost and cost-effectiveness which may be overlapping and could potentially be addressed simultaneously. There was an additional research question in the top 10 about the most effective and cost-effective platforms or services to implement food-based interventions for prevention. Another research question was on the direct, indirect and opportunity costs to families for food-based interventions.

A separate commentary led by GDG members and others involved in the 2023 WHO guideline on evidence gaps related to prevention supports the conduct of effectiveness studies and complementary implementation and cost-effectiveness research.^29^ An additional commentary by GDG members and others involved in the 2023 WHO guideline gives guidance on how to conduct research on resource use and cost-effectiveness for interventions to address wasting and nutritional oedema.^30^

Of note, there were fewer research questions on prevention in the top 20 overall across the different topics and populations. However, many of the research questions prioritised within this CHNRI exercise for prevention of wasting and nutritional oedema were much broader in scope than some of the other questions on management of wasting and nutritional oedema.

#### Application of this research priority agenda to WHO guidance

We hope that this research priority agenda will strengthen the evidence base to better understand wasting and nutritional oedema in infants and children, to potentially update WHO guidelines including the 2023 WHO guideline, and to support implementation of interventions for prevention and management of wasting and nutritional oedema. Such updates should consider the best available evidence that addresses key decision-making domains including but not limited to intervention effectiveness.

Systematic reviews are used to inform WHO recommendations, with the certainty of the evidence based on the GRADE approach, which then impacts the overall certainty of recommendations.^11 12 31^ Reasons that can result in downgrading the evidence according to GRADE include risk of bias, unexplained inconsistency (eg, if subgroups are not well understood), indirectness if studies are not directly applicable or aligned to the question at hand, imprecision which may be an issue if there are too few participants within or across studies, as well as publication bias.^11 12^

There are also other criteria in the GRADE Evidence-to-Decision framework that are crucial to inform recommendations, including resource use and cost-effectiveness, acceptability and feasibility of interventions, and potential impacts on health equity.^11^ There were several research questions prioritised through this CHNRI exercise that are aimed at further understanding these GRADE domains which could directly influence the direction, strength and certainty of WHO recommendations.^11 31^

Overall, this research priority agenda has the potential to harmonise research on key areas, which could in turn increase the certainty of the evidence that underpins recommendations. In addition to this research priority agenda, we have developed core outcome sets for wasting and nutritional oedema which should be applied for effectiveness trials in these populations to further standardise research.^32^ Researchers who are conducting clinical trials should also follow WHO guidance on best practices to ensure that they are conducting ethical and high-quality research in these areas.^33^ However, the developers of the CHNRI exercise have noted that specific research methodologies, such as randomised controlled trials, are not typically mentioned in research priority agendas developed through the CHNRI method since they are ‘unlikely to be answered by a single well-defined study’.^15^

Furthermore, it is important to note that there can be a distinction between research questions and guideline questions, although there is often alignment. In this CHNRI exercise, criteria that participants used to prioritise research questions were answerability, effectiveness, deliverability and effects on equity.^15 16^ The GDG for the 2023 WHO guideline prioritised guideline questions based on three criteria: uncertainty or controversy about best practice, availability or absence of guidance, and impact on health outcomes.^10 34^ Answering research questions in this research priority agenda does not guarantee that findings will be used directly for guidance. However, the likelihood of informing recommendations increases when new, high-quality evidence meaningfully strengthens the body of evidence for a relevant guideline question and/or demonstrates impact on relevant outcomes with sufficient certainty across available studies.

### Limitations

There are several limitations to this CHNRI exercise which we acknowledge, the first being the limitation of the surveys being done in English. As described above, this was an expedited process considering the need to rapidly produce a research priority agenda while nutrition funding has been reduced drastically.^8^ We understand that this may have affected the research questions that were prioritised, and we urge policy makers, researchers, research institutions and funders to consider what research needs to be done to address needs in their settings.

We also acknowledge that there was a relatively small sample size for this CHNRI exercise. However, we took multiple actions to support and promote responses. We circulated the surveys to a large representative group, including members of the GDG, observers and systematic review team leads for the 2023 WHO guideline, the UNICEF/WHO Technical Advisory Group on Wasting and Nutritional Oedema (Acute Malnutrition), attendees of a 2024 research consultation on wasting and nutritional oedema, and WHO regional offices and their networks. We also posted both surveys publicly on en-net (https://www.en-net.org/). Participants who completed survey 1 were also sent survey 2. We sent multiple reminders to the above people and extended deadlines for the two surveys.

A series of analyses by Yoshida *et al*^35^ of 91 people who participated in a CHNRI exercise showed that collective opinion stabilises quite quickly, with a high degree of reproducibility of research priority questions with 45–55 participants.^35^ While we were below this number of participants, the top research questions based on RPSs were characterised by high AEA, with a strong correlation between RPS and AEA indicating expert agreement between participants.

The CHNRI method is a metrics-based approach using surveys which has some inherent limitations, including that it can be an intensive process for participants with a considerable number of research questions to score even within the individual populations and topics, which can make it more challenging to obtain responses as described above. Completing these surveys required a steady internet connection which could realistically be another barrier limiting equitable participation. These challenges may have led to a low response rate and self-selection bias,^35^ the effects of which would be unknown in terms of impact on the prioritised research questions.

Most participants who completed survey 2 identified themselves as researchers. The analyses by Yoshida *et al*^35^ demonstrated that there was overlap for 10 of the top 20 research ideas in a CHNRI exercise between researchers and non-researchers. They identify that there is a more considerable difference between participants based in ‘high-income countries’ versus ‘low-income and middle-income countries’, with an overlap for 8 out of 20 research ideas.^35^ The authors state that it would be useful to do further analysis to answer questions about the impacts of missing responses as well as about the differences based on ‘the number of experts, the diversity of experts, the number of research ideas, or the content and diversity of research ideas’.^35^ Furthermore, CHNRI exercises do not typically involve discussion or consensus among participants to agree on the research priorities after the surveys have been completed. However, this approach allows for each participant’s opinion to be weighted equally without influence from others.

Another key limitation is that patients or caregivers/mothers of infants and children with wasting and/or nutritional oedema were not included in this CHNRI process. We strongly believe that it would be important to conduct further participatory research, which we did not have the resources to do, to ensure that the research questions being addressed reflect the needs of communities and individuals affected by wasting and nutritional oedema. As stated earlier, we urge policy makers, researchers and others to strongly consider what from this agenda is most relevant in their contexts.

## Conclusions

This research priority agenda developed using a CHNRI process aims to facilitate linkages between research, guideline recommendations and implementation. It includes the research questions deemed by participants in this CHNRI process to be critical to address based on their answerability, effectiveness, deliverability and effect on equity, as part of a systematic process. We plan to be responsive to emerging evidence that can be used to inform the 2023 WHO guideline on the prevention and management of wasting and nutritional oedema in infants and children. We will also aim to update this research priority agenda after its conclusion in 2030, and when new goals are set to address wasting and nutritional oedema in infants and children globally.

## Supplementary material

10.1136/bmjgh-2025-021214 online supplemental file 1

10.1136/bmjgh-2025-021214 online supplemental file 2

## Data Availability

All data relevant to the study are included in the article or uploaded as supplementary information.
