# Supplementary material for: A WHO global research priority agenda for wasting and nutritional oedema in infants and children under 5 years
Source: BMJ Glob Health. 2026 Mar 23;10(Suppl 5):e021214. doi: 10.1136/bmjgh-2025-021214 (PMC13034320; doi:10.1136/bmjgh-2025-021214)
Supplement: online supplemental file 1 [file bmjgh-10-Suppl_5-s001.docx]

|  | **Questions** | **Answers** |
| --- | --- | --- |
| **Study conceptualization** | 1. How does this study address local research and policy priorities? 2. How were local researchers involved in study design? | This CHNRI process builds on the development of the 2023 WHO guideline on the prevention and management of wasting and nutritional oedema (acute malnutrition) in infants and children under 5 years, which is a global guideline that provides evidence-informed recommendations and good practice statements to strengthen the care of infants and children with a key focus on health equity.  The research priority agenda described in this paper was done with the goal of strengthening the evidence around wasting and nutritional oedema globally. We aimed to involve a representative group of experts in the prevention and management of wasting and nutritional oedema to participate in this CHNRI process. These could include any researchers, clinicians, policy makers, program implementers, and community partners with expertise in wasting and nutritional oedema. |
| **Research management** | 1. How has funding been used to support the local research team(s)? | We have highlighted this as a limitation in the discussion section of the paper, in the context of describing that in the future we would hope to include patients or caregivers/mothers of infants and children with wasting and/or nutritional oedema. We also state that we strongly believe that it would be important to conduct further participatory research, which we did not have the resources to do, to ensure that the research questions being addressed reflect the needs of communities and individuals affected by wasting and nutritional oedema. As stated earlier, we urge policy makers, researchers, and others to strongly consider what from this agenda is most relevant in their contexts. |
| **Data acquisition and analysis** | 1. How are research staff who conducted data collection acknowledged? 2. How have members of the research partnership been provided with access to study data? 3. How were data used to develop analytical skills within the partnership? | This research did not involve data collection by a research team. Instead, it was a CHNRI process conducted virtually with the involvement of any experts globally who are involved in the prevention and management of wasting and nutritional oedema. |
| **Data interpretation** | 1. How have research partners collaborated in interpreting study data? | This was a process that we led and implemented using a systematic CHNRI process with two online surveys. The first survey specifically inviting participants to add any additional priority research questions that had not already been included. They could add research questions if they felt that these additional questions would have the potential to enhance understanding of wasting and nutritional oedema and/or could inform policy. There was no limit to the number of research questions that could be proposed by participants.  Participants could also provide feedback or suggestions to improve the clarity or framing of any research questions including specific suggestions for wording changes, additional context, or reorganization of the research questions. |
| **Drafting and revising for intellectual content** | 1. How were research partners supported to develop writing skills? 2. How will research products be shared to address local needs? | Research partners were not involved in this CHNRI process that we led which was not the same as primary research.  We plan to share the research priority agenda widely and to get feedback from a variety of interest holders to improve the agenda in the future. |
| **Authorship** | 1. How is the leadership, contribution and ownership of this work by LMIC researchers recognised within the authorship? 2. How have early career researchers across the partnership been included within the authorship team? 3. How has gender balance been addressed within the authorship? | Two authors of this paper are based in LMICs, and another author is from a LMIC but working at WHO in Geneva, Switzerland from an underrepresented group. The first author would be considered early career. The author team includes four people who identify as female and two who identify as male. |
| **Training** | 1. How has the project contributed to training of LMIC researchers? | This project has not contributed to training of LMIC researchers per se, but we hope that this research priority agenda can support researchers in LMICs which was a key objective of this work. |
| **Infrastructure** | 1. How has the project contributed to improvements in local infrastructure? | As described above, in this paper, we share a global research priority agenda to harmonize research across settings and contexts. |
| **Governance** | 1. What safeguarding procedures were used to protect local study participants and researchers? | We consulted with the WHO Research Ethics Review Committee to determine whether informed consent was required, which was not the case since it did not meet WHO’s criteria for research with human participants. |
