# Supplementary material for: A WHO global research priority agenda for wasting and nutritional oedema in infants and children under 5 years
Source: BMJ Glob Health. 2026 Mar 23;10(Suppl 5):e021214. doi: 10.1136/bmjgh-2025-021214 (PMC13034320; doi:10.1136/bmjgh-2025-021214)
Supplement: online supplemental file 2 [file bmjgh-10-Suppl_5-s002.docx]

**A WHO global research priority agenda for wasting and nutritional oedema in infants and children under 5 years**

***Supplemental file***

Allison I Daniel, Jaden Bendabenda, Michael McCaul, Celeste E Naude, Marina Adrianopoli, Zita Weise Prinzo

**Survey 1**

The WHO Steering Committee and methodologists for the 2023 WHO guideline on wasting and nutritional oedema are leading a CHNRI exercise to develop a comprehensive global research priority agenda around wasting and nutritional oedema in infants and children under 5 years. This research priority agenda will aim to address the most urgent evidence and research gaps related to child health and health equity in these populations. The timeline of this agenda will extend through to 2030, in line with the Sustainable Development Goals, which include wasting indicators as part of target 2.2, and the Global Nutrition Targets, which also include wasting and have been extended to 2030.

**Populations and topics of interest:**

1. Infants less than 6 months of age at risk of poor growth and development
2. Infants and children 6-59 months of age with severe wasting and/or nutritional oedema
3. Infants and children 6-59 months of age with moderate wasting
4. Prevention of wasting and nutritional oedema

**Geographic limits:**global

**Time scale:**results of the research expected by 2030

**Research questions from the 2023 WHO guideline**

We compiled the entire list of research needs that emerged from Guideline Development Group (GDG) discussions during the guideline development process for the 2023 WHO guideline on the prevention and management of wasting and nutritional oedema. We converted these to research questions (rather than research needs) for this CHNRI exercise and categorized them by the above populations and topics. We also further organized them by research sub-domains, adapted by Lelijveld et al, 2023 for nutrition, including:

- descriptive (research to assess the burden of the problem, its determinants, and effectiveness of interventions to address the problem)
- delivery (research to improve how nutrition interventions are delivered, financed, and taken-up)
- development (research to improve nutrition interventions that already exist)
- discovery (research that leads to innovation i.e., entirely new nutrition interventions).

**Survey 1 objectives**

- To identify potential research questions that will enhance understanding of wasting and nutritional oedema and have the potential inform policy and practice
- To ensure the list of research questions is comprehensive and clear
- To collect expert feedback and suggestions for improvement

**What you need to do for survey 1**

We first ask you to answer five brief questions about you as a participant (gender, location, role/profession/expertise, and organization type). After this, we ask you to do the following with respect to the research questions:

1)     Review the research questions

- Carefully review the proposed research questions in each section
- Consider whether these questions address critical research gaps and/or have the potential to inform policy and practice

2)     Propose additional research questions (optional)

- If you believe an important research question is missing, you can propose additional questions
- Ensure that any additional questions align with the overall goal of improving understanding and/or addressing gaps in research related to wasting and nutritional oedema

3)     Provide comments (optional)

- Share feedback or suggestions to improve the clarity or framing of any research questions
- Specific suggestions for wording changes, additional context, or reorganization are welcome

**Deadline for submission of survey 1: 16 March 2025 (extended to 20 March 2025)**

**We request that you still submit the survey even if you have no additional proposed research questions or comments.**

**Next steps: survey 2**

After survey 1, we will invite participants to complete survey 2. This will involve scoring all research questions according to four CHNRI criteria: answerability, effectiveness, deliverability, and the effect on equity.

Please note that your responses will be kept anonymous and separate from your email address, and your participation is completely voluntary.

Thank you so much for your contributions to this important process.

If you have any questions, please contact XXXX

**Participant information**

**Gender**

- Woman
- Man
- Non-binary
- Other
- Prefer not to answer

**Primary location (WHO region)**

- African Region
- Region of the Americas
- South-East Asian Region
- European Region
- Eastern Mediterranean Region
- Western Pacific Region

**Primary location (country)**

__________________________

**Role/profession/expertise**

- Researcher
- Clinician
- Policy maker
- Program implementer
- Community partner
- Other

**Organization type**

- Academic/research institution
- Health facility
- Health ministry
- UN agency
- Non-governmental organization
- Community-based organization
- Independent
- Other

**What you need to do for survey 1**

1)     Review the research questions

- Carefully review the proposed research questions in each section
- Consider whether these questions address critical research gaps and/or have the potential to inform policy and practice

2)     Propose additional research questions (optional)

- If you believe an important research question is missing, you can propose additional questions
- Ensure that any additional questions align with the overall goal of improving understanding and/or addressing gaps in research related to wasting and nutritional oedema

3)     Provide comments (optional)

- Share feedback or suggestions to improve the clarity or framing of any research questions
- Specific suggestions for wording changes, additional context, or reorganization are welcome

**The research questions can be found at the document link below, which we suggest you open in a new tab to avoid having to complete the survey again:**[**WHO-global-research-priority-agenda-survey1-Feb2025.pdf**](https://worldhealthorg-my.sharepoint.com/:b:/g/personal/adaniel_who_int/EZ4OQm82lgBFgGqB4O8zFMQBW0N27ZhRMPsoJceeXfmQnA?e=AqDww3)

**Do you have additional research questions to propose that have not yet been included?**

- Yes
- No

**If yes, please share additional proposed research questions**

__________________________________________________________________________________________________________________________________________________________________________________________________________________________________________

**Do you have specific comments about the proposed research questions?**

- Yes
- No

**If yes, please share specific comments on the research questions**

__________________________________________________________________________________________________________________________________________________________________________________________________________________________________________

You have reached the end of survey 1. **Please click done to submit your responses.**Note that we request that you still submit the survey even if you have no additional proposed research questions or comments.

**A WHO global research priority agenda for wasting and nutritional oedema in infants and children under 5 years**

**Survey 2**

The WHO Steering Committee and methodologists for the 2023 WHO guideline on wasting and nutritional oedema are leading a CHNRI exercise to develop a comprehensive global research priority agenda around wasting and nutritional oedema in infants and children under 5 years. This research priority agenda will aim to address the most urgent evidence and research gaps related to child health and health equity in these populations. The timeline of this agenda will extend through to 2030, in line with the Sustainable Development Goals, which include wasting indicators as part of target 2.2, and the Global Nutrition Targets, which also include wasting and have been extended to 2030.

**Populations and topics of interest:**

- Infants less than 6 months of age at risk of poor growth and development
- Infants and children 6-59 months of age with severe wasting and/or nutritional oedema
- Infants and children 6-59 months of age with moderate wasting
- Prevention of wasting and nutritional oedema

**Geographic limits:**global

**Time scale:**results of the research expected by 2030

**Survey 1 (completed)**

Survey 1 was undertaken to ensure the list of research questions is comprehensive and clear for scoring within survey 2. Only unique research questions that were within scope for the populations and topics of interest were added to the list of questions for scoring in survey 2.

It is not a requirement to have completed survey 1 to participate in survey 2.

**Survey 2 objective**

- To score research questions based on their answerability, effectiveness, deliverability, and effects on equity

**What you need to do for survey 2**

1) Select the population(s) and topic(s) for which you will score each research question based on your expertise

2) Review each research question

- Read each research question carefully, reflecting on its importance and relevance to the identified population(s) and topic(s) of interest

3) Score each research question

- Use the four CHNRI criteria below to evaluate and score each research question:
  - Answerability: A research study (qualitative and/or quantitative) to answer this research question is feasible and ethical, and can be done by 2030
  - Effectiveness: The findings of this research (qualitative and/or quantitative) could lead to effective interventions, programs, or policies that improve child health
  - Deliverability: The findings of this research (qualitative and/or quantitative) could lead to interventions, programs, or policies that can realistically be implemented
  - Effect on equity: The findings of this research (qualitative and/or quantitative) could contribute to reducing health inequities among disadvantaged children
- For each criterion, you may select agree, neither agree nor disagree, disagree, or don’t know

**Deadline for submission of survey 2: 11 May 2025 **extended to 25 May 2025***

Please note that your responses will be kept anonymous and your participation is completely voluntary.

Thank you so much for your contributions to this important process.

If you have any questions, please contact XXXX

**Participant information**

**Gender**

- Woman
- Man
- Non-binary
- Other
- Prefer not to answer

**Primary location (WHO region)**

- African Region
- Region of the Americas
- South-East Asian Region
- European Region
- Eastern Mediterranean Region
- Western Pacific Region

**Primary location (country)**

__________________________

**Role/profession/expertise**

- Researcher
- Clinician
- Policy maker
- Program implementer
- Community partner
- Other

**Organization type**

- Academic/research institution
- Health facility
- Health ministry
- UN agency
- Non-governmental organization
- Community-based organization
- Independent
- Other

**Would you like to score research questions for infants less than 6 months of age at risk of poor growth and development (33 questions)?**

- Yes
- No

**Infants less than 6 months of age at risk of poor growth and development (33 questions)**

1) What screening criteria should be used to detect infants less than 6 months of age at risk of poor growth and development in the community?^[[1]](#footnote-1)^

|  | Agree | Neither agree nor disagree | Disagree | Don't know |
| --- | --- | --- | --- | --- |
| Answerability: Research studies (qualitative and/or quantitative) to answer this research question are feasible and ethical, and can be done by 2030 |  |  |  |  |
| Effectiveness: The findings of this research (qualitative and/or quantitative) could lead to effective interventions, programs, or policies that improve child health |  |  |  |  |
| Deliverability: The findings of this research (qualitative and/or quantitative) could lead to interventions, programs, or policies that can realistically be implemented |  |  |  |  |
| Effect on equity: The findings of this research (qualitative and/or quantitative) could contribute to reducing health inequities among disadvantaged children |  |  |  |  |

2) How can infants less than 6 months of age at risk of poor growth and development be identified across settings?

3) What are the most appropriate methods and tools for assessing breastfeeding, including whether an infant less than 6 months of age is being exclusively breastfed, having difficulties breastfeeding, etc.?

4) What are the factors affecting resilience and cognitive development of infants less than 6 months of age at risk of poor growth and development?

5) What are the optimal and feasible enrolment and monitoring/transfer criteria to best identify infants less than 6 months of age at risk of poor growth and development across different settings?

6) What are the impacts of services and how these services are delivered focusing on infants less than 6 months of age at risk of poor growth and development?

7) What are the drivers of early cessation of breastfeeding in infants less than 6 months of age at risk of poor growth and development and what could be done to prevent early cessation?

8) What are the drivers of the use of prelacteal feeds in infants less than 6 months of age at risk of poor growth and development?

9) How can it be determined whether breastmilk production is below an infant’s needs at different ages up to 6 months of age?

10) What are reliable criteria for defining optimal growth in infants less than 6 months of age at risk of poor growth and development?

11) What is the need for and the effectiveness of supplemental milk and breastmilk fortifiers predominantly in inpatient settings for infants less than 6 months of age at risk of poor growth and development, as well as those with severe wasting and/or nutritional oedema specifically?

12) What is the effectiveness of different types of formulas including F-75, diluted F-100, full-strength F-100, and infant formulas (potentially consider pre-term formulas if appropriate) as well as donor human milk in inpatient settings for infants less than 6 months of age at risk of poor growth and development, as well as those with severe wasting and/or nutritional oedema specifically?

13) What is the effectiveness of different types of formulas in infants less than 6 months of age at risk of poor growth and development who have already stopped exclusive breastfeeding?

14) Could complementary foods or therapeutic foods be introduced while infants at risk of poor growth and development are less than 6 months of age?

15) What is the effectiveness of routine amoxicillin and other antibiotics for infants less than 6 months of age at risk of poor growth and development?

16) What is the effectiveness of probiotic supplementation for infants less than 6 months of age at risk of poor growth and development?

17) What prioritization criteria can be used for infants less than 6 months of age at risk of poor growth and development if caseloads are high and resources limited?

18) What are optimal minimum standards for monitoring and improving quality of interventions for infants less than 6 months of age at risk of poor growth and development?

19) Does inpatient care for infants less than 6 months at risk of poor growth and development improve outcomes compared to outpatient care, based on a set of criteria or different factors?

20) What approaches are most effective to re-establish breastfeeding for mothers of infants less than 6 months of age at risk of poor growth and development who have stopped breastfeeding?

21) Which tools and strategies can health workers use to assess and manage simple breastfeeding problems effectively?

22) What are the cadres, training needs, and tools that can most effectively support breastfeeding?

23) What are the acceptability and existing practices around wet nursing, supplementary suckling technique, re-establishment of breastfeeding, etc.?

24) How should infants less than 6 months of age at risk of poor growth and development be best linked with related services (e.g., Integrated Management of Newborn and Childhood Illness, immunizations, growth monitoring, mental health, disability treatment services)?

25) What are the most effective approaches to ensure that infants less than 6 months of age at risk of poor growth and development are breastfed in critical situations?

26) How should infants less than 6 months of age at risk of poor growth and development who fail to respond to initial supported breastfeeding and clinical treatment be managed?

27) Which packages of care are most effective at improving outcomes in infants less than 6 months of age at risk of poor growth and development and preventing wasting and nutritional oedema in these infants?

28) What is the effectiveness of interventions targeting social, psychological, and economic challenges faced by mothers/caregivers of infants less than 6 months of age at risk of poor growth and development?

29) What intervention packages to support existing health staff, such as using peer counsellors for the assessment and management of breastfeeding/lactation difficulties exist and how do they compare?

30) What is the feasibility of implementation and effectiveness of interventions aimed at addressing maternal mental health among mothers /caregivers of infants less than 6 months of age at risk of poor growth and development?

31) What are the priority medical and nutritional support and interventions for mothers/caregivers of infants less than 6 months of age at risk of poor growth and development?

32) How can maternal interventions be improved to enhance breastmilk quality and quantity for infants less than 6 months of age at risk of poor growth and development?

33) What are the pathways underlying mortality in infants less than 6 months of age at risk of poor growth and development?

**Would you like to score research questions for infants and children 6-59 months of age with severe wasting and/or nutritional oedema (62 questions)?**

- Yes
- No

**Infants and children 6-59 months of age with severe wasting and/or nutritional oedema (62 questions)**

1) What are social factors that may be associated with the risk of poor outcomes in children with severe wasting and/or nutritional oedema?^[[2]](#footnote-2)^

|  | Agree | Neither agree nor disagree | Disagree | Don't know |
| --- | --- | --- | --- | --- |
| Answerability: Research studies (qualitative and/or quantitative) to answer this research question are feasible and ethical, and can be done by 2030 |  |  |  |  |
| Effectiveness: The findings of this research (qualitative and/or quantitative) could lead to effective interventions, programs, or policies that improve child health |  |  |  |  |
| Deliverability: The findings of this research (qualitative and/or quantitative) could lead to interventions, programs, or policies that can realistically be implemented |  |  |  |  |
| Effect on equity: The findings of this research (qualitative and/or quantitative) could contribute to reducing health inequities among disadvantaged children |  |  |  |  |

2) What is the pathophysiology of severe wasting and nutritional oedema?

3) What is the relationship between wasting and stunting?

4) What are the specific causes of nutritional oedema?

5) Should management of nutritional oedema differ from management of severe wasting?

6) How can health workers differentiate between nutritional causes of oedema and other underlying conditions when detected in a community setting?

7) What pathogens (e.g. cryptosporidium) drive diarrhea-related mortality in hospitalized children with severe wasting and/or nutritional oedema, and how can they be addressed?

8) What chronic conditions are associated with non-response or treatment failure in infants and children with severe wasting and/or nutritional oedema?

9) What are the risk factors (individual, household, community, program-level) for relapse to severe wasting and/or nutritional oedema?

10) What is the effectiveness of social interventions, psychosocial support, and mental health for mothers/caregivers of in infants and children with severe wasting and/or nutritional oedema?

11) Which interventions are effective to prevent relapse and improve long-term health and development outcomes in infants and children with severe wasting and/or nutritional oedema?

12) Can SQ-LNS prevent or reduce relapse to severe wasting and/or nutritional oedema?

13) What are the most appropriate methods and tools for assessing and supporting breastfeeding, including relactation where needed and possible for infants and children over 6 months and up to 2 years?

14) What are the factors affecting resilience and cognitive development of infants and children who have experienced severe wasting and/or nutritional oedema?

15) What are the early markers of likelihood of non-recovery from severe wasting and/or nutritional oedema?

16) How should hydration status in infants and children with severe wasting and/or nutritional oedema be assessed and classified?

17) What is the optimal management strategy infants and children severe wasting and/or nutritional oedema and with dehydration, circulatory impairment, and shock?

18) What is the effectiveness of ReSoMal compared to low-osmolarity ORS without added potassium in infants and children with severe wasting and/or nutritional oedema?

19) What are the optimal standardized criteria of feeding intolerance in infants and children with severe wasting and/or nutritional oedema?

20) What is the prevalence of feeding intolerance to therapeutic milks and lactose intolerance in infants and children with severe wasting and/or nutritional oedema in inpatient settings?

21) What is the prevalence of refeeding syndrome and optimal management of refeeding syndrome in infants and children with severe wasting and/or nutritional oedema in inpatient settings?

22) What are the optimal compositions of F-75, F-100, and RUTF for severe wasting and/or nutritional oedema?

23) What is the effectiveness of different novel (e.g. low or no-milk) ready-to-use therapeutic food (RUTF) in the management of severe wasting and/or nutritional oedema?

24) Can adding potassium and/or sodium to F-75 achieve optimal content for infants and children with severe wasting and/or nutritional oedema?

25) How can F-75 be revised to best meet the needs of children with nutritional oedema?

26) What other electrolyte imbalances affect hospitalized children with severe wasting and/or nutritional oedema?

27) What are the effects of donor human milk and hydrolyzed and lactose-free feeds for infants and children with severe wasting and/or nutritional oedema who are not tolerating F-75 or F-100?

28) Does urine specific gravity help determine the hydration status of infants and children with severe wasting and/or nutritional oedema who presented with dehydration?

29) What are the energy/nutrient requirements of infants and children with severe wasting and/or nutritional oedema, including when children improve from severe to moderate wasting?

30) What is the optimal rate of weight gain in infants and children with severe wasting and/or nutritional oedema?

31) What are effective interventions (e.g. dietary, other) that can be provided in the absence of ready-to-use therapeutic food (RUTF) for the management of severe wasting and/or nutritional oedema?

32) What are the long-term cardiometabolic effects and other consequences of ready-to-use therapeutic food (RUTF)?

33) What are the cost and cost-effectiveness of different quantities of ready-to-use therapeutic food (RUTF)?

34) How can infants and children with severe wasting and/or nutritional oedema and underlying medical conditions and/or disability be managed?

35) What are effective, safe, and culturally adaptable treatment strategies for managing severe wasting and/or nutritional oedema in infants and children with concurrent metabolic disorders or food allergies (e.g., lactose intolerance, Phenylketonuria)?

36) What is the optimal antibiotic regimen for infants and children with wasting and/or nutritional oedema?

37) Does inpatient care of children with severe wasting and/or nutritional oedema with specific risk factors improve outcomes compared to outpatient care?

38) What are potential risk-targeted follow-up strategies of infants and children with severe wasting and/or nutritional oedema?

39) Should a renal function test be a requirement before starting therapeutic milks in infants and children with severe wasting and/or nutritional oedema?

40) What is the effectiveness of different ready-to-use therapeutic food (RUTF) protocol options with reducing quantities compared to standard quantities?

41) For infants and children with severe wasting and/or nutritional oedema who are growing but do not reach the recovery within the maximum length of stay for a program, is it feasible for them to be kept longer under treatment?

42) What are the maximal duration and optimal duration that treatment should take for severe wasting and/or nutritional oedema?

43) What field-friendly, low-cost tools for screening, identification, and referral can be used to best support infants and children with severe wasting and/or nutritional oedema who are not responding to treatment?

44) What is the effectiveness of community health workers (CHWs) in management of severe wasting and/or nutritional oedema throughout the care pathway?

45) What is the cost-effectiveness of community health workers (CHWs) for management of severe wasting and/or nutritional oedema and the impacts of this approach on coverage and other services?

46) What tools, support systems, etc. can be utilized to maximize effectiveness of community health workers (CHWs) management?

47) What are implementation considerations for psychosocial stimulation including intensity and frequency, as well as who can provide the intervention and in what contexts, for infants and children with severe wasting and/or nutritional oedema?

48) What are appropriate methods and tools for monitoring and improving early childhood development in primary and secondary care for infants less than 6 months of age at risk of poor growth and development?

49) What are the cost and cost-effectiveness of alternative ready-to-use therapeutic food (RUTF) formulations in different settings?

50) What is the optimal quantity and packaging of ready-to-use therapeutic food (RUTF) from a usage/user perspective?

51) What are the cost and cost-effectiveness of psychosocial stimulation interventions for infants and children with severe wasting and/or nutritional oedema, including the costs for caregivers and trained staff?

52) What is the cost-effectiveness of cash transfers (based on metrics such as disability-adjusted life years) and the impact of this intervention in different settings for mothers/caregivers of infants and children with severe wasting and/or nutritional oedema?

53) What are appropriate methods and tools for monitoring and improving early child development in primary and secondary care for infants and children with severe wasting and/or nutritional oedema?

54) What are the effectiveness and cost-effectiveness of weekly versus fortnightly visits during management of severe wasting and/or nutritional oedema?

55) What interventions/approaches increase treatment coverage for infants and children with severe wasting and/or nutritional oedema?

56) What is the quality adjusted coverage/effective coverage of management of wasting and nutritional oedema?

57) What are the effects of combining psychosocial stimulation with other interventions in infants and children with severe wasting and/or nutritional oedema?

58) What is the impact of post-exit cash transfers combined with individual counselling for mothers/caregivers of infants and children with severe wasting and/or nutritional oedema?

59) What is the efficacy of antibiotics (apart from daily oral co-trimoxazole prophylaxis) with different durations provided to infants and children with severe wasting and/or nutritional oedema as post-discharge interventions?

60) What are the effects of different integrated post-exit packages in infants and children with severe wasting and/or nutritional oedema?

61) What are the pathways underlying mortality in infants and children with severe wasting and/or nutritional oedema?

62) Can somatic hydrolysis be used in existing F-75 formulas to create a hydrolyzed F-75 for infants and children with severe wasting and/or nutritional oedema?

**Would you like to score research questions for infants and children 6-59 months of age with moderate wasting (29 questions)?**

- Yes
- No

**Infants and children 6-59 months of age with moderate wasting (29 questions)**

1) How should hydration status in infants and children with moderate wasting be assessed and classified?^[[3]](#footnote-3)^

|  | Agree | Neither agree nor disagree | Disagree | Don't know |
| --- | --- | --- | --- | --- |
| Answerability: Research studies (qualitative and/or quantitative) to answer this research question are feasible and ethical, and can be done by 2030 |  |  |  |  |
| Effectiveness: The findings of this research (qualitative and/or quantitative) could lead to effective interventions, programs, or policies that improve child health |  |  |  |  |
| Deliverability: The findings of this research (qualitative and/or quantitative) could lead to interventions, programs, or policies that can realistically be implemented |  |  |  |  |
| Effect on equity: The findings of this research (qualitative and/or quantitative) could contribute to reducing health inequities among disadvantaged children |  |  |  |  |

2) What is the pathophysiology of moderate wasting?

3) What are the risk factors (individual, household, community, program-level) for relapse to moderate wasting?

4) What are the energy/nutrient requirements of infants and children with moderate wasting?

5) What is the optimal rate of weight gain in infants and children with moderate wasting?

6) Which interventions are effective to prevent relapse and improve long-term health and development outcomes in infants and children with moderate wasting?

7) Can SQ-LNS prevent or reduce relapse to moderate wasting?

8) What is the effectiveness of different approaches to dietary management of moderate wasting, including the use of available home foods in different contexts?

9) What is the response to interventions in infants and children with moderate wasting who have specific risk factors?

10) Would infants and children with moderate wasting who do not meet criteria for prioritization with specially formulated foods (SFFs) in low-risk settings benefit from a dietary intervention?

11) What are effective, safe, and culturally adaptable treatment strategies for managing moderate wasting in infants and children with concurrent metabolic disorders or food allergies (e.g., lactose intolerance, Phenylketonuria)?

12) What is the optimal antibiotic regimen for infants and children with moderate wasting?

13) Which infants and children with moderate wasting should be prioritized for supplementation based on risk factors?

14) What is the effectiveness of community health workers (CHWs) in management of moderate wasting throughout the care pathway?

15) What is the cost-effectiveness of community health workers (CHWs) for management of moderate wasting and the impacts of this approach on coverage and other services?

16) What tools, support systems, etc. can be utilized to maximize effectiveness of community health workers (CHWs) management?

17) What are implementation considerations for psychosocial stimulation including intensity and frequency, as well as who can provide the intervention and in what contexts, for infants and children with moderate wasting?

18) What are the cost and cost-effectiveness of psychosocial stimulation interventions for infants and children with moderate wasting, including the costs for caregivers and trained staff?

19) What are the cost and cost-effectiveness of specially formulated foods (SFFs) and other dietary interventions?

20) What are the cost and cost-effectiveness of managing moderate wasting using available home foods in different contexts?

21) What is the feasibility of reaching all infants and children with moderate wasting who require specially formulated foods (SFFs) in a variety of contexts?

22) What are the long-term effects of different types and durations of specially formulated foods (SFFs)?

23) What are the best approaches to develop and implement context-specific interventions using home-based foods/interventions, apart from specially formulated foods (SFFs), for the management of moderate wasting?

24) What are the most effective and cost-effective targeting strategies for the provision of specially formulated foods (SFFs) for moderate wasting in humanitarian settings?

25) What are appropriate methods and tools for monitoring and improving early child development in primary and secondary care for infants and children with moderate wasting?

26) What is the cost-effectiveness of integrating moderate wasting into the primary heath care system (in places where moderate wasting is managed outside the health system)?

27) What are the effects of combining psychosocial stimulation with other interventions in infants and children moderate wasting?

28) What is the optimal micronutrient content of specially formulated foods (SFFs)?

29) What are the pathways underlying mortality in infants and children with moderate wasting?

**Would you like to score research questions for prevention of wasting and nutritional oedema (26 questions)?**

- Yes
- No

**Prevention of wasting and nutritional oedema (26 questions)**

1) What are the most effective interventions in humanitarian contexts to prevent wasting and nutritional oedema?^[[4]](#footnote-4)^

|  | Agree | Neither agree nor disagree | Disagree | Don't know |
| --- | --- | --- | --- | --- |
| Answerability: Research studies (qualitative and/or quantitative) to answer this research question are feasible and ethical, and can be done by 2030 |  |  |  |  |
| Effectiveness: The findings of this research (qualitative and/or quantitative) could lead to effective interventions, programs, or policies that improve child health |  |  |  |  |
| Deliverability: The findings of this research (qualitative and/or quantitative) could lead to interventions, programs, or policies that can realistically be implemented |  |  |  |  |
| Effect on equity: The findings of this research (qualitative and/or quantitative) could contribute to reducing health inequities among disadvantaged children |  |  |  |  |

2) What are the impacts of specially formulated foods (SFFs) for prevention of wasting and nutritional oedema on body composition, neurodevelopment, and long-term health outcomes?

3) What are potential adverse effects of specially formulated foods (SFFs) for prevention of wasting and nutritional oedema, including displacing breastfeeding and home diets, encouraging consumption of processed foods, and displacing healthy food production and preparation skills?

4) What are the potential impacts of maternal interventions in the context of prevention of wasting and nutritional oedema?

5) Which community, household and child-level factors associated with likelihood of progressing to wasting or nutritional oedema?

6) What are the impacts of maternal, infant and young child feeding (IYCF) counselling/interventions on improved practices and on the prevention of wasting and nutritional oedema?

7) What is the effectiveness of other types of prevention interventions apart from SQ-LNS and MQ-LNS for prevention of wasting and nutritional oedema?

8) What is the effectiveness of social protection programs and approaches (e.g. maternity/paternity leaves; cash support; financial compensation for parental leaves) for prevention of wasting and nutritional oedema?

9) What is the potential role of cash programming in prevention of wasting and nutritional oedema, in isolation or in combination with other interventions?

10) What are the impacts of preventive interventions on equity in different contexts?

11) How is the relationship between household food insecurity and wasting and nutritional oedema?

12) Are different preventive strategies needed for infants and children who are 6-23 months of age compared to children who are 24-59 months of age?

13) What is the optimal duration and timing of preventive interventions to prevent wasting and nutritional oedema?

14) What are the optimal quantity, duration and timing of food-based interventions for prevention of wasting and nutritional oedema?

15) What are the most effective and cost-effective household and geographical targeting criteria to implement preventive specially formulated foods (SFFs) for prevention of wasting and nutritional oedema?

16) What is the cost-effectiveness of interventions for prevention of wasting and nutritional oedema, including logistics and implementation costs?

17) What are the most effective and cost-effective platforms or services to implement food-based interventions for prevention of wasting and nutritional oedema?

18) What are the direct, indirect, and opportunity costs to families of food-based interventions for prevention of wasting and nutritional oedema?

19) What is the optimal delivery of maternal, infant and young child feeding (IYCF) counselling/interventions and the types/models of counselling that are most effective for prevention of wasting and nutritional oedema?

20) What is the effectiveness of maternal, infant and young child feeding (IYCF) counselling/interventions on prevention of wasting and nutritional oedema in areas with different levels of wasting and food insecurity?

21) What are the cost and cost-effectiveness of preventive interventions for wasting and nutritional oedema compared to each other?

22) What is the feasibility and sustainability of blanket compared to targeted approaches across settings for prevention of wasting and nutritional oedema?

23) What would be the impact of interventions that combine nutrition interventions (e.g. SFFs) with interventions for prevention/management of infections (e.g. malaria, respiratory infections, diarrheal disease), for both prevention and management of wasting and nutritional oedema?

24) How can caregivers be better supported in providing home-based meals that prevent wasting and nutritional oedema?

25) What low-cost and scalable livelihood initiatives can build resilience against wasting and nutritional oedema?

26) What are the effects of a package of interventions for the prevention of wasting and nutritional oedema that includes maternal and infant and young child feeding (IYCF) counselling?

Thank you so much for your time and effort to complete this survey.

**Scores for all research questions**

| **Research question** | **RPS** | **AEA** | **Population** |
| --- | --- | --- | --- |
| What is the effectiveness of different ready-to-use therapeutic food (RUTF) protocol options with reducing quantities compared to standard quantities? | 93.9 | 89.3 | Severe |
| What are potential risk-targeted follow-up strategies of infants and children with severe wasting and/or nutritional oedema? | 93.6 | 89.5 | Severe |
| What are reliable criteria for defining optimal growth in infants less than 6 months of age at risk of poor growth and development? | 92.8 | 86.3 | Infants |
| What are the cost and cost-effectiveness of specially formulated foods (SFFs) and other dietary interventions? | 92.5 | 88.7 | Moderate |
| What are the most effective interventions in humanitarian contexts to prevent wasting and nutritional oedema? | 92.1 | 87.1 | Prevention |
| What is the cost-effectiveness of integrating moderate wasting into the primary heath care system (in places where moderate wasting is managed outside the health system)? | 92.0 | 89.3 | Moderate |
| What tools, support systems, etc. can be utilized to maximize effectiveness of community health workers (CHWs) management? | 91.9 | 86.8 | Severe |
| Which infants and children with moderate wasting should be prioritized for supplementation based on risk factors? | 91.8 | 87.4 | Moderate |
| What are the cost and cost-effectiveness of different quantities of ready-to-use therapeutic food (RUTF)? | 91.8 | 88.1 | Severe |
| Which interventions are effective to prevent relapse and improve long-term health and development outcomes in infants and children with moderate wasting? | 91.7 | 87.1 | Moderate |
| What are the cost and cost-effectiveness of preventive interventions for wasting and nutritional oedema compared to each other? | 91.5 | 84.6 | Prevention |
| Which tools and strategies can health workers use to assess and manage simple breastfeeding problems effectively? | 91.5 | 85.7 | Infants |
| Would infants and children with moderate wasting who do not meet criteria for prioritization with specially formulated foods (SFFs) in low-risk settings benefit from a dietary intervention? | 91.4 | 83.6 | Moderate |
| What intervention packages to support existing health staff, such as using peer counsellors for the assessment and management of breastfeeding/lactation difficulties exist and how do they compare? | 91.2 | 84.7 | Infants |
| What are the most effective and cost-effective targeting strategies for the provision of specially formulated foods (SFFs) for moderate wasting in humanitarian settings? | 90.7 | 83.7 | Moderate |
| What is the effectiveness of different types of formulas including F-75, diluted F-100, full-strength F-100, and infant formulas (potentially consider pre-term formulas if appropriate) as well as donor human milk in inpatient settings for infants less than 6 months of age at risk of poor growth and development, as well as those with severe wasting and/or nutritional oedema specifically? | 90.7 | 84.9 | Infants |
| What are the cost and cost-effectiveness of managing moderate wasting using available home foods in different contexts? | 90.6 | 85.0 | Moderate |
| How should infants less than 6 months of age at risk of poor growth and development who fail to respond to initial supported breastfeeding and clinical treatment be managed? | 90.5 | 83.2 | Infants |
| Which interventions are effective to prevent relapse and improve long-term health and development outcomes in infants and children with severe wasting and/or nutritional oedema? | 90.1 | 84.8 | Severe |
| What is the cost-effectiveness of interventions for prevention of wasting and nutritional oedema, including logistics and implementation costs? | 90.0 | 83.0 | Prevention |
| What is the effectiveness of community health workers (CHWs) in management of moderate wasting throughout the care pathway? | 89.8 | 84.8 | Moderate |
| What are the most effective and cost-effective platforms or services to implement food-based interventions for prevention of wasting and nutritional oedema? | 89.5 | 81.2 | Prevention |
| What is the potential role of cash programming in prevention of wasting and nutritional oedema, in isolation or in combination with other interventions? | 89.5 | 83.3 | Prevention |
| What are the risk factors (individual, household, community, program-level) for relapse to moderate wasting? | 89.2 | 81.4 | Moderate |
| What chronic conditions are associated with non-response or treatment failure in infants and children with severe wasting and/or nutritional oedema? | 89.2 | 82.1 | Severe |
| What are the cost and cost-effectiveness of alternative ready-to-use therapeutic food (RUTF) formulations in different settings? | 89.0 | 80.4 | Severe |
| What low-cost and scalable livelihood initiatives can build resilience against wasting and nutritional oedema? | 89.0 | 82.0 | Prevention |
| What are the risk factors (individual, household, community, program-level) for relapse to severe wasting and/or nutritional oedema? | 88.9 | 83.2 | Severe |
| What screening criteria should be used to detect infants less than 6 months of age at risk of poor growth and development in the community? | 88.8 | 82.2 | Infants |
| Which packages of care are most effective at improving outcomes in infants less than 6 months of age at risk of poor growth and development and preventing wasting and nutritional oedema in these infants? | 88.8 | 84.6 | Infants |
| What tools, support systems, etc. can be utilized to maximize effectiveness of community health workers (CHWs) management? | 88.6 | 82.0 | Moderate |
| What is the effectiveness of other types of prevention interventions apart from SQ-LNS and MQ-LNS for prevention of wasting and nutritional oedema? | 88.6 | 77.9 | Prevention |
| What is the effectiveness of community health workers (CHWs) in management of severe wasting and/or nutritional oedema throughout the care pathway? | 88.6 | 80.9 | Severe |
| What is the cost-effectiveness of community health workers (CHWs) for management of severe wasting and/or nutritional oedema and the impacts of this approach on coverage and other services? | 88.6 | 80.9 | Severe |
| What is the effectiveness of different approaches to dietary management of moderate wasting, including the use of available home foods in different contexts? | 88.5 | 79.9 | Moderate |
| What are the acceptability and existing practices around wet nursing, supplementary suckling technique, re-establishment of breastfeeding, etc.? | 88.5 | 81.8 | Infants |
| What prioritization criteria can be used for infants less than 6 months of age at risk of poor growth and development if caseloads are high and resources limited? | 88.4 | 81.2 | Infants |
| What is the effectiveness of social interventions, psychosocial support, and mental health for mothers/caregivers of in infants and children with severe wasting and/or nutritional oedema? | 88.4 | 83.2 | Severe |
| What is the cost-effectiveness of community health workers (CHWs) for management of moderate wasting and the impacts of this approach on coverage and other services? | 88.3 | 81.1 | Moderate |
| What are the cost and cost-effectiveness of psychosocial stimulation interventions for infants and children with severe wasting and/or nutritional oedema, including the costs for caregivers and trained staff? | 88.3 | 82.0 | Severe |
| What are the priority medical and nutritional support and interventions for mothers/caregivers of infants less than 6 months of age at risk of poor growth and development? | 87.9 | 80.7 | Infants |
| What is the feasibility of implementation and effectiveness of interventions aimed at addressing maternal mental health among mothers /caregivers of infants less than 6 months of age at risk of poor growth and development? | 87.8 | 79.8 | Infants |
| What is the need for and the effectiveness of supplemental milk and breastmilk fortifiers predominantly in inpatient settings for infants less than 6 months of age at risk of poor growth and development, as well as those with severe wasting and/or nutritional oedema specifically? | 87.5 | 80.3 | Infants |
| What are the best approaches to develop and implement context-specific interventions using home-based foods/interventions, apart from specially formulated foods (SFFs), for the management of moderate wasting? | 87.5 | 80.3 | Moderate |
| What are the effectiveness and cost-effectiveness of weekly versus fortnightly visits during management of severe wasting and/or nutritional oedema? | 87.4 | 79.4 | Severe |
| What are the optimal quantity, duration and timing of food-based interventions for prevention of wasting and nutritional oedema? | 87.4 | 80.7 | Prevention |
| What are the energy/nutrient requirements of infants and children with severe wasting and/or nutritional oedema, including when children improve from severe to moderate wasting? | 86.9 | 78.5 | Severe |
| What are appropriate methods and tools for monitoring and improving early childhood development in primary and secondary care for infants less than 6 months of age at risk of poor growth and development? | 86.8 | 80.0 | Severe |
| What interventions/approaches increase treatment coverage for infants and children with severe wasting and/or nutritional oedema? | 86.8 | 80.6 | Severe |
| What is the effectiveness of different novel (e.g. low or no-milk) ready-to-use therapeutic food (RUTF) in the management of severe wasting and/or nutritional oedema? | 86.7 | 78.9 | Severe |
| How can infants less than 6 months of age at risk of poor growth and development be identified across settings? | 86.7 | 76.1 | Infants |
| What are the direct, indirect, and opportunity costs to families of food-based interventions for prevention of wasting and nutritional oedema? | 86.6 | 74.7 | Prevention |
| What are effective interventions (e.g. dietary, other) that can be provided in the absence of ready-to-use therapeutic food (RUTF) for the management of severe wasting and/or nutritional oedema? | 86.6 | 82.1 | Severe |
| Can SQ-LNS prevent or reduce relapse to moderate wasting? | 86.5 | 79.7 | Moderate |
| What pathogens (e.g. cryptosporidium) drive diarrhea-related mortality in hospitalized children with severe wasting and/or nutritional oedema, and how can they be addressed? | 86.5 | 81.7 | Severe |
| For infants and children with severe wasting and/or nutritional oedema who are growing but do not reach the recovery within the maximum length of stay for a program, is it feasible for them to be kept longer under treatment? | 86.3 | 78.6 | Severe |
| What are the potential impacts of maternal interventions in the context of prevention of wasting and nutritional oedema? | 86.0 | 78.6 | Prevention |
| What is the optimal rate of weight gain in infants and children with severe wasting and/or nutritional oedema? | 85.9 | 80.6 | Severe |
| What is the impact of post-exit cash transfers combined with individual counselling for mothers/caregivers of infants and children with severe wasting and/or nutritional oedema? | 85.8 | 78.7 | Severe |
| What are the cadres, training needs, and tools that can most effectively support breastfeeding? | 85.8 | 75.8 | Infants |
| What are the early markers of likelihood of non-recovery from severe wasting and/or nutritional oedema? | 85.8 | 76.7 | Severe |
| What is the optimal antibiotic regimen for infants and children with wasting and/or nutritional oedema? | 85.8 | 77.9 | Severe |
| What are the energy/nutrient requirements of infants and children with moderate wasting? | 85.7 | 79.2 | Moderate |
| What is the effectiveness of social protection programs and approaches (e.g. maternity/paternity leaves; cash support; financial compensation for parental leaves) for prevention of wasting and nutritional oedema? | 85.7 | 78.7 | Prevention |
| Can SQ-LNS prevent or reduce relapse to severe wasting and/or nutritional oedema? | 85.7 | 78.3 | Severe |
| What is the effectiveness of interventions targeting social, psychological, and economic challenges faced by mothers/caregivers of infants less than 6 months of age at risk of poor growth and development? | 85.2 | 78.1 | Infants |
| Should management of nutritional oedema differ from management of severe wasting? | 85.2 | 80.8 | Severe |
| What is the cost-effectiveness of cash transfers (based on metrics such as disability-adjusted life years) and the impact of this intervention in different settings for mothers/caregivers of infants and children with severe wasting and/or nutritional oedema? | 85.1 | 76.3 | Severe |
| What approaches are most effective to re-establish breastfeeding for mothers of infants less than 6 months of age at risk of poor growth and development who have stopped breastfeeding? | 84.8 | 75.3 | Infants |
| What are the drivers of early cessation of breastfeeding in infants less than 6 months of age at risk of poor growth and development and what could be done to prevent early cessation? | 84.8 | 76.0 | Infants |
| What is the optimal micronutrient content of specially formulated foods (SFFs)? | 84.7 | 76.6 | Moderate |
| What are the effects of different integrated post-exit packages in infants and children with severe wasting and/or nutritional oedema? | 84.6 | 75.7 | Severe |
| How should infants less than 6 months of age at risk of poor growth and development be best linked with related services (e.g., Integrated Management of Newborn and Childhood Illness, immunizations, growth monitoring, mental health, disability treatment services)? | 84.5 | 75.3 | Infants |
| What would be the impact of interventions that combine nutrition interventions (e.g. SFFs) with interventions for prevention/management of infections (e.g. malaria, respiratory infections, diarrheal disease), for both prevention and management of wasting and nutritional oedema? | 84.5 | 72.8 | Prevention |
| What is the optimal management strategy infants and children severe wasting and/or nutritional oedema and with dehydration, circulatory impairment, and shock? | 84.4 | 78.4 | Severe |
| What are the most appropriate methods and tools for assessing breastfeeding, including whether an infant less than 6 months of age is being exclusively breastfed, having difficulties breastfeeding, etc.? | 84.4 | 73.4 | Infants |
| What are the effects of combining psychosocial stimulation with other interventions in infants and children moderate wasting? | 84.3 | 76.4 | Moderate |
| What are the pathways underlying mortality in infants less than 6 months of age at risk of poor growth and development? | 84.0 | 71.8 | Infants |
| What is the effectiveness of ReSoMal compared to low-osmolarity ORS without added potassium in infants and children with severe wasting and/or nutritional oedema? | 83.9 | 76.8 | Severe |
| What field-friendly, low-cost tools for screening, identification, and referral can be used to best support infants and children with severe wasting and/or nutritional oedema who are not responding to treatment? | 83.8 | 78.2 | Severe |
| What are the effects of a package of interventions for the prevention of wasting and nutritional oedema that includes maternal and infant and young child feeding (IYCF) counselling? | 83.7 | 75.5 | Prevention |
| How can maternal interventions be improved to enhance breastmilk quality and quantity for infants less than 6 months of age at risk of poor growth and development? | 83.6 | 73.7 | Infants |
| Which community, household and child-level factors associated with likelihood of progressing to wasting or nutritional oedema? | 83.5 | 74.1 | Prevention |
| What are the maximal duration and optimal duration that treatment should take for severe wasting and/or nutritional oedema? | 83.3 | 76.8 | Severe |
| What are the most effective and cost-effective household and geographical targeting criteria to implement preventive specially formulated foods (SFFs) for prevention of wasting and nutritional oedema? | 83.3 | 75.6 | Prevention |
| How can caregivers be better supported in providing home-based meals that prevent wasting and nutritional oedema? | 83.3 | 73.0 | Prevention |
| Are different preventive strategies needed for infants and children who are 6-23 months of age compared to children who are 24-59 months of age? | 82.8 | 73.0 | Prevention |
| What are the effects of combining psychosocial stimulation with other interventions in infants and children with severe wasting and/or nutritional oedema? | 82.8 | 73.4 | Severe |
| Does inpatient care for infants less than 6 months at risk of poor growth and development improve outcomes compared to outpatient care, based on a set of criteria or different factors? | 82.6 | 74.9 | Infants |
| What is the optimal rate of weight gain in infants and children with moderate wasting? | 82.6 | 76.5 | Moderate |
| What are potential adverse effects of specially formulated foods (SFFs) for prevention of wasting and nutritional oedema, including displacing breastfeeding and home diets, encouraging consumption of processed foods, and displacing healthy food production and preparation skills? | 82.5 | 78.0 | Prevention |
| How can F-75 be revised to best meet the needs of children with nutritional oedema? | 82.5 | 74.7 | Severe |
| How can infants and children with severe wasting and/or nutritional oedema and underlying medical conditions and/or disability be managed? | 82.3 | 73.0 | Severe |
| What are the impacts of maternal, infant and young child feeding (IYCF) counselling/interventions on improved practices and on the prevention of wasting and nutritional oedema? | 82.2 | 75.5 | Prevention |
| What is the response to interventions in infants and children with moderate wasting who have specific risk factors? | 82.0 | 71.6 | Moderate |
| What are the optimal compositions of F-75, F-100, and RUTF for severe wasting and/or nutritional oedema? | 81.9 | 74.3 | Severe |
| What are the pathways underlying mortality in infants and children with severe wasting and/or nutritional oedema? | 81.8 | 72.7 | Severe |
| What are implementation considerations for psychosocial stimulation including intensity and frequency, as well as who can provide the intervention and in what contexts, for infants and children with moderate wasting? | 81.7 | 71.8 | Moderate |
| What is the optimal duration and timing of preventive interventions to prevent wasting and nutritional oedema? | 81.7 | 68.6 | Prevention |
| What are social factors that may be associated with the risk of poor outcomes in children with severe wasting and/or nutritional oedema? | 81.6 | 72.6 | Severe |
| What is the prevalence of refeeding syndrome and optimal management of refeeding syndrome in infants and children with severe wasting and/or nutritional oedema in inpatient settings? | 81.5 | 71.1 | Severe |
| What are the pathways underlying mortality in infants and children with moderate wasting? | 81.5 | 71.6 | Moderate |
| What are the specific causes of nutritional oedema? | 81.4 | 74.3 | Severe |
| How should hydration status in infants and children with severe wasting and/or nutritional oedema be assessed and classified? | 81.3 | 75.8 | Severe |
| What are the optimal and feasible enrolment and monitoring/transfer criteria to best identify infants less than 6 months of age at risk of poor growth and development across different settings? | 81.3 | 69.9 | Infants |
| What are the drivers of the use of prelacteal feeds in infants less than 6 months of age at risk of poor growth and development? | 81.3 | 70.6 | Infants |
| What is the quality adjusted coverage/effective coverage of management of wasting and nutritional oedema? | 81.0 | 72.1 | Severe |
| What is the optimal antibiotic regimen for infants and children with moderate wasting? | 80.9 | 72.3 | Moderate |
| What are implementation considerations for psychosocial stimulation including intensity and frequency, as well as who can provide the intervention and in what contexts, for infants and children with severe wasting and/or nutritional oedema? | 80.8 | 73.7 | Severe |
| What are the cost and cost-effectiveness of psychosocial stimulation interventions for infants and children with moderate wasting, including the costs for caregivers and trained staff? | 80.7 | 69.7 | Moderate |
| What is the optimal quantity and packaging of ready-to-use therapeutic food (RUTF) from a usage/user perspective? | 80.7 | 73.2 | Severe |
| Does inpatient care of children with severe wasting and/or nutritional oedema with specific risk factors improve outcomes compared to outpatient care? | 80.6 | 73.4 | Severe |
| What are the impacts of services and how these services are delivered focusing on infants less than 6 months of age at risk of poor growth and development? | 80.6 | 67.4 | Infants |
| What are the effects of donor human milk and hydrolyzed and lactose-free feeds for infants and children with severe wasting and/or nutritional oedema who are not tolerating F-75 or F-100? | 80.5 | 74.2 | Severe |
| What is the effectiveness of different types of formulas in infants less than 6 months of age at risk of poor growth and development who have already stopped exclusive breastfeeding? | 80.2 | 71.8 | Infants |
| What is the feasibility and sustainability of blanket compared to targeted approaches across settings for prevention of wasting and nutritional oedema? | 79.6 | 66.8 | Prevention |
| What is the relationship between wasting and stunting? | 79.3 | 67.9 | Severe |
| Can adding potassium and/or sodium to F-75 achieve optimal content for infants and children with severe wasting and/or nutritional oedema? | 79.2 | 72.0 | Severe |
| What is the efficacy of antibiotics (apart from daily oral co-trimoxazole prophylaxis) with different durations provided to infants and children with severe wasting and/or nutritional oedema as post-discharge interventions? | 79.0 | 70.6 | Severe |
| What are the factors affecting resilience and cognitive development of infants less than 6 months of age at risk of poor growth and development? | 79.0 | 62.4 | Infants |
| What is the optimal delivery of maternal, infant and young child feeding (IYCF) counselling/interventions and the types/models of counselling that are most effective for prevention of wasting and nutritional oedema? | 78.9 | 67.7 | Prevention |
| What are optimal minimum standards for monitoring and improving quality of interventions for infants less than 6 months of age at risk of poor growth and development? | 78.9 | 66.8 | Infants |
| What other electrolyte imbalances affect hospitalized children with severe wasting and/or nutritional oedema? | 78.6 | 66.6 | Severe |
| What are the optimal standardized criteria of feeding intolerance in infants and children with severe wasting and/or nutritional oedema? | 78.1 | 68.4 | Severe |
| Could complementary foods or therapeutic foods be introduced while infants at risk of poor growth and development are less than 6 months of age? | 77.7 | 71.2 | Infants |
| What are the most appropriate methods and tools for assessing and supporting breastfeeding, including relactation where needed and possible for infants and children over 6 months and up to 2 years? | 77.5 | 69.5 | Severe |
| What are the most effective approaches to ensure that infants less than 6 months of age at risk of poor growth and development are breastfed in critical situations? | 77.3 | 63.8 | Infants |
| What is the effectiveness of routine amoxicillin and other antibiotics for infants less than 6 months of age at risk of poor growth and development? | 77.1 | 65.7 | Infants |
| Does urine specific gravity help determine the hydration status of infants and children with severe wasting and/or nutritional oedema who presented with dehydration? | 77.1 | 66.1 | Severe |
| What are effective, safe, and culturally adaptable treatment strategies for managing severe wasting and/or nutritional oedema in infants and children with concurrent metabolic disorders or food allergies (e.g., lactose intolerance, Phenylketonuria)? | 77.0 | 66.5 | Severe |
| What is the feasibility of reaching all infants and children with moderate wasting who require specially formulated foods (SFFs) in a variety of contexts? | 76.6 | 67.3 | Moderate |
| What are appropriate methods and tools for monitoring and improving early child development in primary and secondary care for infants and children with severe wasting and/or nutritional oedema? | 76.5 | 69.4 | Severe |
| What are appropriate methods and tools for monitoring and improving early child development in primary and secondary care for infants and children with moderate wasting? | 76.3 | 64.7 | Moderate |
| What is the effectiveness of maternal, infant and young child feeding (IYCF) counselling/interventions on prevention of wasting and nutritional oedema in areas with different levels of wasting and food insecurity? | 76.2 | 67.5 | Prevention |
| What is the prevalence of feeding intolerance to therapeutic milks and lactose intolerance in infants and children with severe wasting and/or nutritional oedema in inpatient settings? | 75.9 | 61.9 | Severe |
| Can somatic hydrolysis be used in existing F-75 formulas to create a hydrolyzed F-75 for infants and children with severe wasting and/or nutritional oedema? | 75.5 | 66.2 | Severe |
| How is the relationship between household food insecurity and wasting and nutritional oedema? | 75.1 | 67.2 | Prevention |
| What are effective, safe, and culturally adaptable treatment strategies for managing moderate wasting in infants and children with concurrent metabolic disorders or food allergies (e.g., lactose intolerance, Phenylketonuria)? | 75.0 | 61.0 | Moderate |
| What are the long-term effects of different types and durations of specially formulated foods (SFFs)? | 74.4 | 65.0 | Moderate |
| What are the impacts of specially formulated foods (SFFs) for prevention of wasting and nutritional oedema on body composition, neurodevelopment, and long-term health outcomes? | 74.2 | 65.9 | Prevention |
| What are the factors affecting resilience and cognitive development of infants and children who have experienced severe wasting and/or nutritional oedema? | 73.4 | 56.8 | Severe |
| How can it be determined whether breastmilk production is below an infantâ€™s needs at different ages up to 6 months of age? | 72.9 | 59.0 | Infants |
| What is the effectiveness of probiotic supplementation for infants less than 6 months of age at risk of poor growth and development? | 72.5 | 60.0 | Infants |
| What is the pathophysiology of severe wasting and nutritional oedema? | 71.0 | 58.6 | Severe |
| How should hydration status in infants and children with moderate wasting be assessed and classified? | 70.3 | 59.8 | Moderate |
| What are the impacts of preventive interventions on equity in different contexts? | 69.0 | 53.9 | Prevention |
| What are the long-term cardiometabolic effects and other consequences of ready-to-use therapeutic food (RUTF)? | 68.6 | 57.8 | Severe |
| Should a renal function test be a requirement before starting therapeutic milks in infants and children with severe wasting and/or nutritional oedema? | 68.1 | 59.6 | Severe |
| How can health workers differentiate between nutritional causes of oedema and other underlying conditions when detected in a community setting? | 68.1 | 60.4 | Severe |
| What is the pathophysiology of moderate wasting? | 67.6 | 58.2 | Moderate |

AEA, average expert agreement; RPS, research priority score

1. These below four CHNRI criteria are repeated for each individual question but have been intentionally omitted from this document to enhance readability. [↑](#footnote-ref-1)
2. These below four CHNRI criteria are repeated for each individual question but have been intentionally omitted from this document to enhance readability. [↑](#footnote-ref-2)
3. These below four CHNRI criteria are repeated for each individual question but have been intentionally omitted from this document to enhance readability. [↑](#footnote-ref-3)
4. These below four CHNRI criteria are repeated for each individual question but have been intentionally omitted from this document to enhance readability. [↑](#footnote-ref-4)
